# Influenza Neuraminidase Inhibitors: Synthetic Approaches, Derivatives and Biological Activity

**DOI:** 10.3390/molecules21111513

**Published:** 2016-11-11

**Authors:** Pedro Laborda, Su-Yan Wang, Josef Voglmeir

**Affiliations:** Glycomics and Glycan Bioengineering Research Center, College of Food Science and Technology, Nanjing Agricultural University, 1 Weigang, Nanjing 210095, China; pedro.laborda@njau.edu.cn (P.L.); wangsuyan1008@126.com (S.-Y.W.)

**Keywords:** influenza treatment, neuraminidase inhibitors, organic synthesis, total synthesis, sialic acid analogues

## Abstract

Despite being a common viral disease, influenza has very negative consequences, causing the death of around half a million people each year. A neuraminidase located on the surface of the virus plays an important role in viral reproduction by contributing to the release of viruses from infected host cells. The treatment of influenza is mainly based on the administration of neuraminidase inhibitors. The neuraminidase inhibitors zanamivir, laninamivir, oseltamivir and peramivir have been commercialized and have been demonstrated to be potent influenza viral neuraminidase inhibitors against most influenza strains. In order to create more potent neuraminidase inhibitors and fight against the surge in resistance resulting from naturally-occurring mutations, these anti-influenza drugs have been used as templates for the development of new neuraminidase inhibitors through structure-activity relationship studies. Here, we review the synthetic routes to these commercial drugs, the modifications which have been performed on these structures and the effects of these modifications on their inhibitory activity.

## 1. Introduction

Influenza is a serious viral illness which can lead to hospitalization and death, especially in the elderly [1,2,3]. Influenza spreads around the world in yearly outbreaks, resulting in about three to five million cases of severe illness and approximately 250,000 to 500,000 deaths [3,4]. Newly mutated forms of the flu virus appear every year and some of them show high levels of resistance to the standard antiviral drugs [5,6,7]. Furthermore, during the last century four influenza pandemics have occurred: “Spanish influenza” in 1918, “Asian influenza” in 1958, “Hong Kong influenza” in 1968, and “avian influenza” in 2004 [8]. Influenza is an RNA virus and is subdivided into three genera: influenza A, influenza B and influenza C. Influenza A is the most virulent virus and has provoked the devastating pandemics of the past. Influenza A can be divided into different serotypes based on the antibody response to these viruses. The most famous serotypes are H1N1, which caused the “Spanish influenza” and the 2009 pandemic, H2N2, which caused the “Asian influenza”, H3N2, which caused the “Hong Kong influenza”, and H5N1, which caused the “avian influenza”. Influenza type B and C are much less common than influenza A.

**Figure 1:**
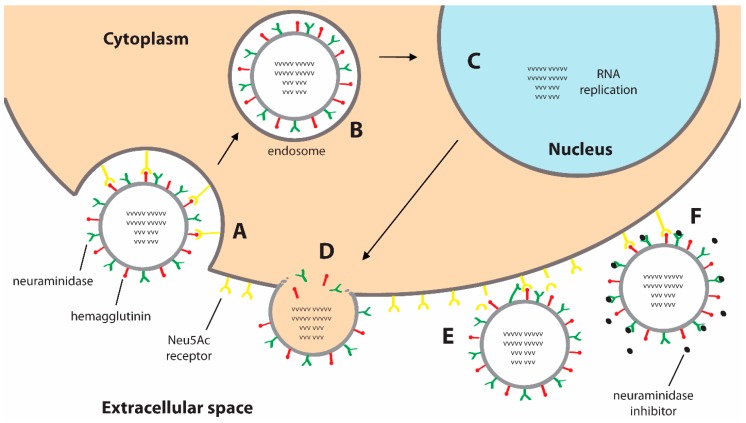
Influenza virus replication pathway and the effect of neuraminidase inhibitors. (**A**) The virus is linked to the host cell via hemagglutinin employing the terminal Neu5Ac residue of the surface receptor. Then, the endocytosis of the virus occurs; (**B**) Viral RNA is released into the cytoplasm where is transferred to the host cell nucleus; (**C**) Viral RNA replication and mRNA synthesis occurs inside the nucleus. The RNA is then released into the cytoplasm; (**D**) Viral proteins are synthetized using mRNA and directed to the cell membrane for virus assembly. After incorporation of the genome fragments, virus budding begins; (**E**) After budding, the virus surface is attached at the Neu5Ac receptor. The neuraminidase is able to hydrolyze Neu5Ac allowing the release of the virus; (**F**) Neuraminidase activity is inhibited in presence of neuraminidase inhibitors preventing the release of the virus and reducing pathogenicity.

The two glycoproteins found on the influenza virus surface envelope are hemagglutinin and neuraminidase (EC 3.2.1.18) (Figure 1) [9,10,11,12]. Hemagglutinin is responsible for viral attachment to the cell surface receptor, which is a terminal sialic acid (*N*-acetylneuraminic acid, Neu5Ac) residue usually linked to a galactose in a α-(2,3) or α-(2,6) glyosidic linkage [9]. The functional neuraminidase, on the other hand, is anchored to the viral membrane by a hydrophobic sequence near the *N*-terminus [13,14,15,16,17]. It has been shown in tissue culture that neuraminidase activity is required to destroy viral receptors by removing the sialic acid of the hemagglutinin-sialic acid linkage, thereby contributing to the release of progeny viruses from infected cells [18,19,20].

**Figure 2:**
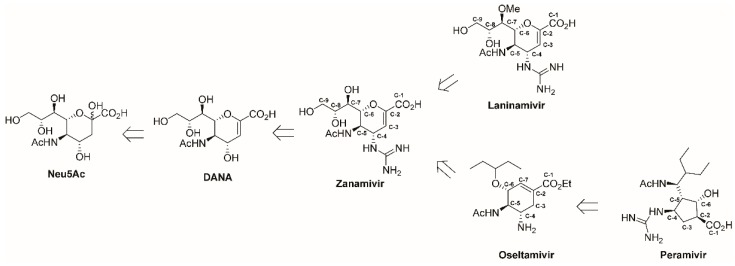
Retrosynthetic analysis of the evolution of influenza neuraminidase inhibitors.

Medical treatment of influenza is generally based on the administration of neuraminidase inhibitors [21,22,23,24,25,26]. 2,3-Didehydro-2-deoxy-*N*-acetylneuraminic acid, also called DANA, was the first influenza neuraminidase inhibitor reported (Figure 2) [27]. While DANA has never been commercialized, its structure has been used as a template for the discovery of inhibitors which are both more potent and better tolerated by humans. Of these, zanamivir, oseltamivir, laninamivir and peramivir have emerged as promising long-acting neuraminidase inhibitors for the treatment and prophylaxis of human influenza virus infection (Figure 2) [28,29,30,31,32,33,34,35]. However, several naturally occurring influenza neuraminidase mutations, such as H274Y mutations in H1N1 and H5N1 influenza A strains, have demonstrated significant resistance towards the above drugs [5,36,37]. For this reason, the development of antiviral drugs which are effective against new strains of influenza virus through the creation of novel influenza neuraminidase inhibitors or by improving the inhibitory activity of existing antiviral drugs is a vibrant research field [38]. Nowadays, the development of new neuraminidase inhibitors is generally based on the synthesis of derivatives of the previously mentioned commercial drugs. Here, we review all the synthetic strategies which have been reported for the production of these compounds, the modifications performed on their structures, and the effects of these modification on their biological activities.

## 2. Zanamivir, Laninamivir and Other Derivatives

Zanamivir, commercialized under the name Relenza, received regulatory approval as a neuraminidase-targeting anti-influenza drug in 1999 [32]. Although originally only suitable for parenteral (subcutaneous or intravenous) administration, zanamivir was developed for oral inhalation, targeting the upper respiratory tract [32]. Zanamivir was derived from DANA by the introduction of a guanidine group linked to C-4 [3,39,40]. This modification resulted in significantly increased inhibitory activity. The following synthetic routes to produce zanamivir have been reported.

### 2.1. Synthesis of Zanamivir

**Scheme 1:**
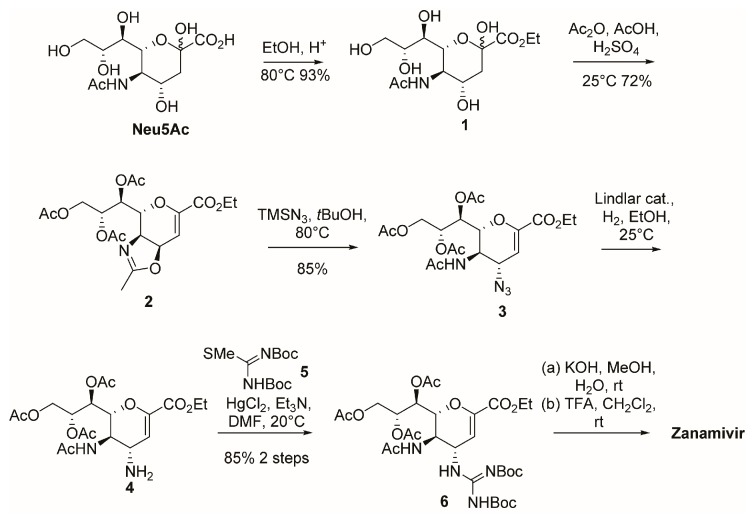
The strategy for the synthesis of zanamivir developed by von Itzstein and coworkers [41].

Zanamivir was first synthesized by von Itzstein and coworkers [41] using sialic acid Neu5Ac as the starting material (Scheme 1). Neu5Ac was first converted to the ethylester **1**, which was treated with acetic anhydride in acetic acid containing a catalytic amount of concentrated sulfuric acid giving **2**. The ring-opening reaction with trimethylsilyl azide occurs by a backside nucleophilic attack. After hydrogenation, the amine product **4** was converted to the guanidine derivative **6** by treatment with 1,3-bis(tert-butoxycarbonyl)-2-methylthiopseudourea (**5**) using HgCl_2_ as the promoter. After saponification and removal of *tert*-butoxycarbonyl (Boc) using trifluoroacetic acid (TFA), zanamivir was isolated in 30%–50% yields. This synthetic strategy was later improved with minor modifications by Scheigetz and coworkers [42] and by Chandler and coworkers [43] allowing the production of zanamivir on a large scale.

**Scheme 2:**
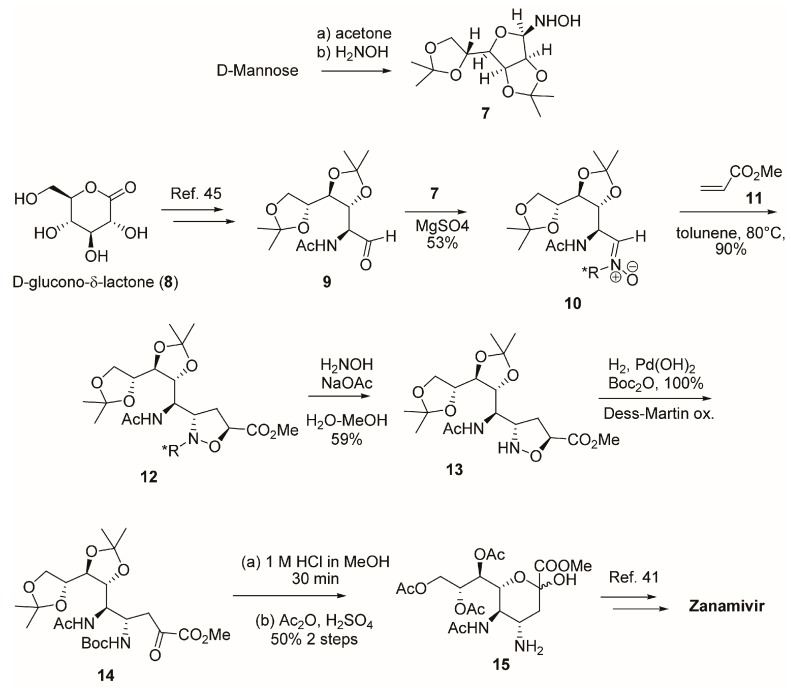
Synthetic route to zanamivir developed by Yao and coworkers [44].

Since 1994, three further synthetic routes for the production of zanamivir have been reported [44,45,46]. Yao and coworkers reported the synthesis of zanamivir using d-glucono-δ-lactone (**8**) as the starting material (Scheme 2) [44], which was easily converted into **9** after protection steps and reduction of the carboxylic acid to aldehyde [47,48]. **9** was allowed to react with chiral hydroxylamine **7**, which was prepared from d-mannose, leading to nitrone **10**. The 1,3-dipolar cycloaddition of **9** with methyl acrylate was accomplished in a stereoselective manner, and the chiral auxiliary (R*) was removed by transamination with hydroxylamine to obtain the isoxazolidine **13**. After hydrogenolysis of the N-O bond and protection as a Boc carbamate, the alcohol group of compound **14** was oxidized using Dess-Martin periodinane. The Boc protecting group was then selectively removed by treatment with 1 M methanolic HCl and the resulting amine was treated with acetic anhydride containing 10% *v*/*v* sulfuric acid and to give compound **15**. The remaining steps were carried out under similar conditions to those reported by von Itzstein and coworkers [41].

**Scheme 3:**
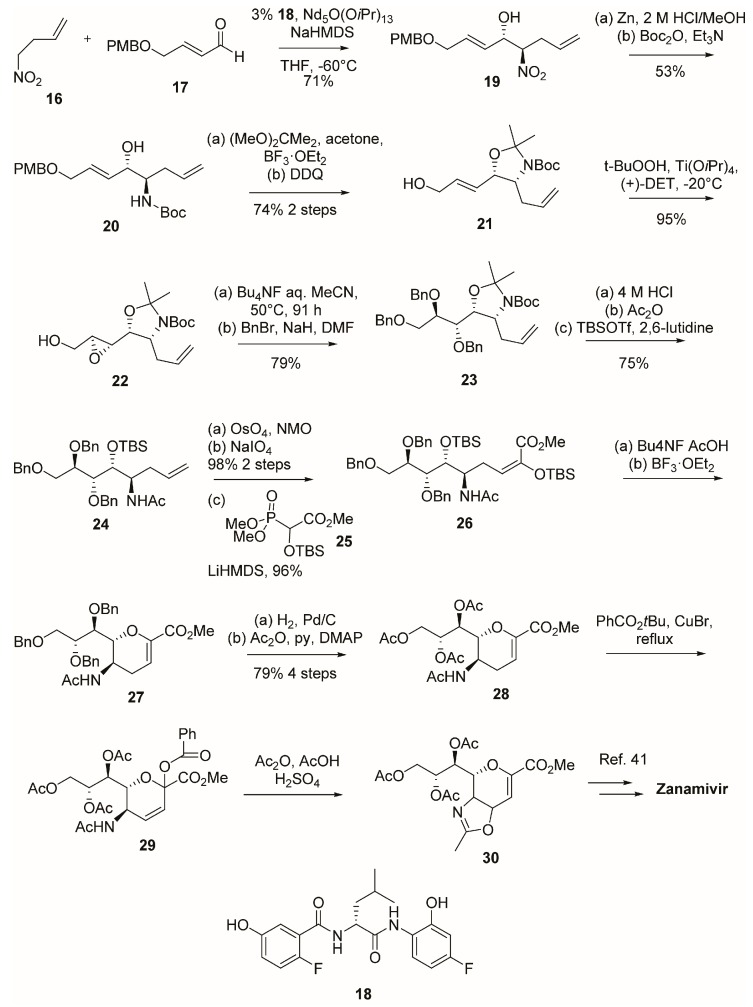
Synthetic strategy to zanamivir developed by Nitabaru and coworkers [45].

Nitabaru, Kumagai and Shibasaki reported a synthesis of zanamivir using (*E*)-4-methoxy-benzyloxy-2-butanal as the starting material (Scheme 3) [45]. An asymmetric Henry reaction between 4-nitro-1-butene (**16**) and (*E*)-4-methoxybenzyloxy-2-butanal (**17**) using an organometallic complex prepared by combining Nd_5_O(O*i*Pr)_13_ and sodium bis(trimethylsilyl)amide (NaHMDS) with chiral ligand **18** was performed to obtain the desired anti-adduct **19** with high enantioselectivity (94% ee). The compound **19** nitro group was then reduced with zinc in the presence of hydrochloric acid, and the amine was protected with Boc to give compound **20**. After protection of the vicinal carbamate and alcohol moieties as an *N*,*O*-acetal and removal of the *p*-methoxybenzyloxy (PMB) protecting group, **21** was subjected to a Katsuki-Sharpless asymmetric allylic epoxidation by treatment with *tert*-butylhydroperoxide, Ti(O*i*Pr)_4_ and (+)-diethyl tartrate (DET) to achieve **22**. Oxirane **22** was then ring-opened by treatment with aqueous tetrabutylammonium fluoride (TBAF) and the resulting alcohols were subjected to perbenzylation. The resulting compound **23** was treated with acid in order to remove the Boc group. Hydrolysis of the oxazolidine, followed by acetylation of the amino group and silylation of the hydroxyl group was carried out to give compound **24**. The terminal double bond of **24** was hydroxylated by treatment with OsO_4_, followed by cleavage with sodium periodate, to give the corresponding aldehyde which was subjected to a Wittig reaction with **25** to afford compound **26**. After deprotection of the tert-butyldimethylsilyl group (TBS) with TBAF-AcOH, **26** was treated with BF_3_·OEt_2_ to obtain tetrahydropyranyl hemiketal **27**. The benzyl groups (Bn) were then removed under hydrogen atmosphere in the presence of Pd/C, and acetic anhydride was used for protection of the hydroxyl groups as acetates. A benzoate group was introduced into the C-2 position of **28** by a copper-mediated oxidation with tert-butyl perbenzoate, giving **29**. The subsequent treatment with H_2_SO_4_/Ac_2_O/AcOH led to oxazoline **30**. The synthesis of zanamivir was completed according to conditions reported by von Itzstein and coworkers [41] in Scheme 1.

**Scheme 4:**
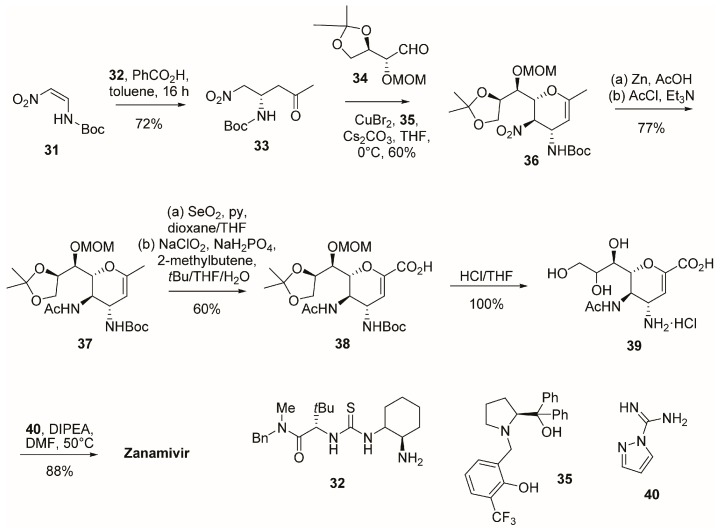
The synthetic route to zanamivir described by Ma and coworkers [46].

Ma and co-workers reported a synthetic route beginning from tert-butyl (2-nitrovinyl)carbamate (**31**) (Scheme 4) [46]. This compound was used as the substrate for a Michael addition reaction with acetone using catalytic amounts of thiourea-based complex **32**, which was previously described by Huang and Jacobsen [49]. Compound **33** was obtained with 98% *ee* and 72% yield. Compound **33** was then subjected to a Henry reaction with aldehyde **34** by treatment with CuBr_2_ in presence of ligand **35** [50]. The nitro group of compound **36** was reduced using Zn/AcOH and then protected with an acetyl group (Ac). SeO_2_ was used for the selective oxidation of C-1 to achieve acid **38**. After deprotection of the methoxymethyl acetal (MOM) and Boc protecting groups by treatment with hydrochloric acid and formation of the guanidine group by addition of compound **39**, zanamivir was obtained with an overall yield of 18%. This strategy was performed on a multigram scale (30 g) demonstrating the potential of this 8-step synthetic route. Although great efforts have been made to enhance the synthetic route of von Itzstein and coworkers [41], both high yields (30%–50%), a low number of synthetic steps (a 6-step route) and the low price of the starting material (Neu5Ac) makes this industrial pathway difficult to improve upon.

### 2.2. C-1 Modifications

**Scheme 5:**
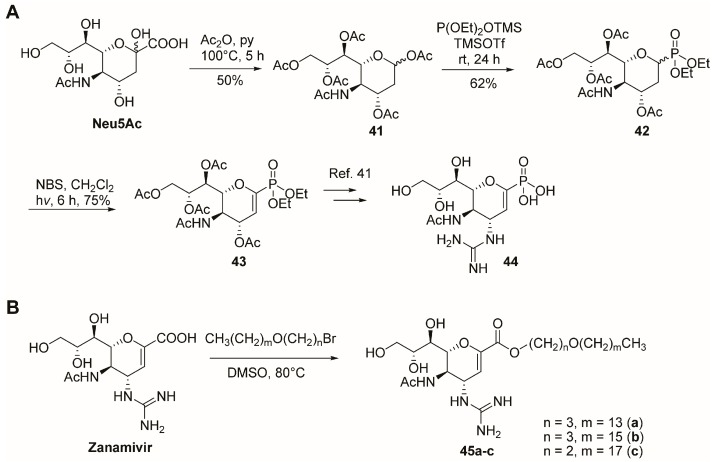
Synthesis of C-1 modified zanamivir analogues. (**A**) Synthetic strategy for zanaphosphor (**44**) developed by Shie and coworkers [52]; (**B**) Synthetic strategy for zanamivir ester derivatives (**45a**–**c**) developed by Li and coworkers [54].

Among the reported modifications to zanamivir, derivatization at the C-1 of the pyranose ring are particularly significant. Both esterification of the carboxylic acid, and the substitution of this functional group for phosphonate have been reported. Vasella and Wyler reported the first synthesis of a phosphonic acid analogue of DANA [51], while, Shie and co-workers later reported the synthesis of zanamivir phosphonate (**44**), also called zanaphosphor, using sialic acid Neu5Ac as the starting material (Scheme 5A) [52]. This sialic acid was protected with acetic anhydride in presence of pyridine (py) at 100 °C, with concomitant decarboxylation to obtain compound **41**. The substitution of the anomeric acetate was carried out using trimethylsilyl diethyl phosphite as the nucleophile and trimethylsilyl trifluoromethylsulfonate (TMSOTf) as a promoter to give the phosphonate compound **42** as a mixture of α and β anomers (2:3). The Dehydration was performed using *N*-bromosuccinimide (NBS) under photochemical conditions to afford compound **43**. Finally, similar conditions as those reported by von Itzstein and co-workers [41] were used for the introduction of the guanidine group at C-4 and deprotection steps. Zanamivir phosphonate showed stronger inhibitory activities against H1N1, H3N2 and H5N1 influenza neuraminidases in comparison with zanamivir and against H1N1 in comparison with oseltamivir. Bren and coworkers have performed binding energy calculations for zanamivir, oseltamivir and peramivir derivatives bearing a C-1 linked sulfonate group in place of the carboxylic acid moiety [53], which predict that zanamivir sulfonate, oseltamivir sulfonate and peramivir sulfonate should all exhibit stronger binding to avian influenza neuraminidase H5N1 than their carboxylate and phosphate analogues.

Li et al. have reported a straightforward methodology for the synthesis of zanamivir alkoxyalkyl ester derivatives **45a**–**c** (Scheme 5B) [54]. This method was based on treating zanamivir with alkoxyalkyl bromides in presence of dimethylsulfoxide (DMSO) and triethyamine at 80 °C. These compounds demonstrated improved bioavaility when orally administered. However, all derivatives displayed lower inhibitory activity against H1N1 and H3N2 influenza neuraminidases in comparison to zanamivir.

### 2.3. C-4 Modifications

**Scheme 6:**
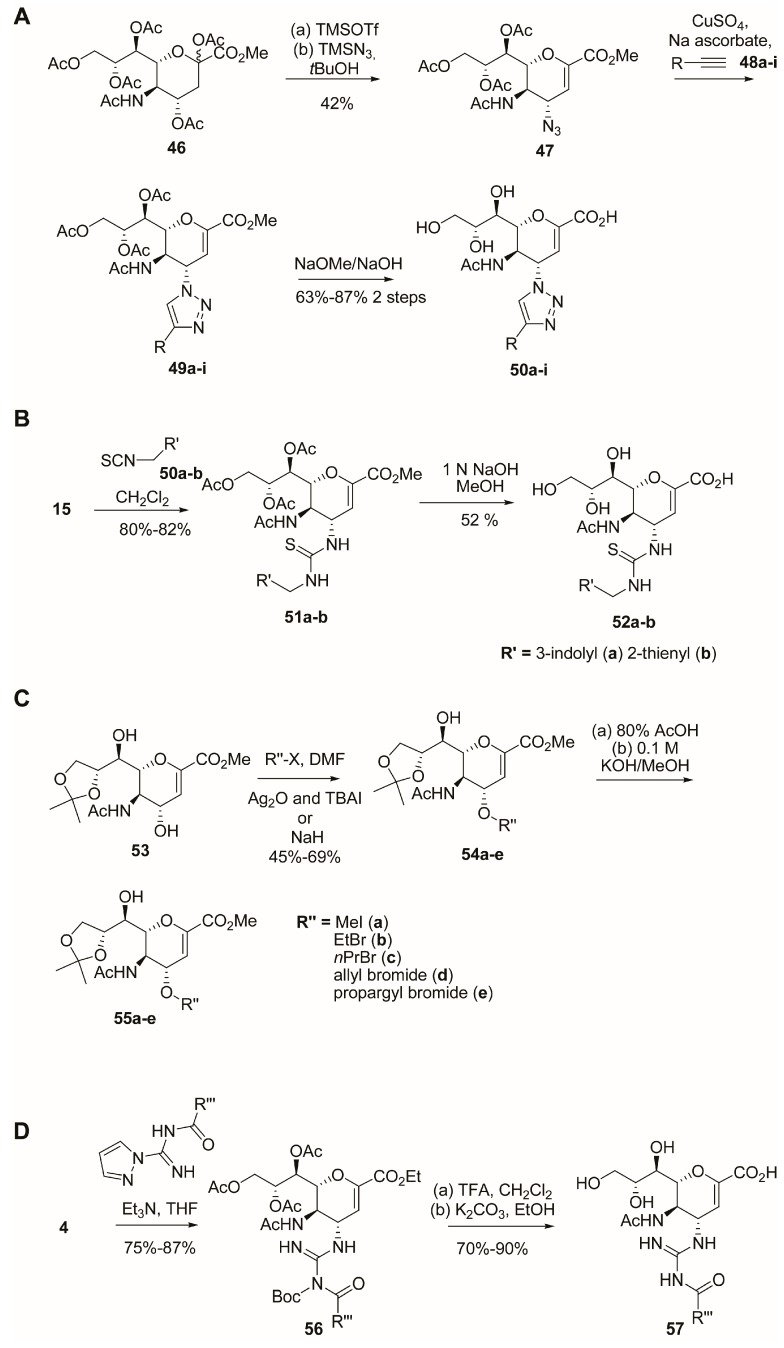
Synthesis of C-4 modified zanamivir analogues bearing triazole groups **50a**–**l** (**A**) [55]; thiocarbamates **52a**,**b** (**B**) [44]; alkyl chains **55a**–**e** (**C**) [57] or acylguanidines **57** (**D**) [58].

Gervay-Hage and Lu reported the synthesis of C-4 triazole analogues of zanamivir **49** (Scheme 6A) [55]. Acetylated Neu5Ac (**46**) was used as the starting material. This compound was treated with TMSOTf followed by reaction with trimethylsylil azide (TMSN_3_) to obtain a zanamivir derivative bearing a C-4 linked azido group (**47**). A series of protected triazole compounds (**49**) were afforded by Cu(I)-catalyzed 1,3-dipolar addition with different alkynes. Finally, deprotection of the acetate groups was carried out using NaOMe/MeOH to produce C-4 triazole zanamivir analogues **50a**–**i**. The same methodology was used by Shen and coworkers for the synthesis of C-4 and C-8 modified zanamivir analogues [56]. Yao and coworkers reported the synthesis of C-4 thiocarbamide derivatives from zanamivir intermediate **15** (Scheme 6B) [44]. A number of different thiocarbamates reacted smoothly with **15** to afford the corresponding carbamides **51a** and **51b**. The acetyl deprotection was carried out by treatment with 1 N NaOH solution in methanol to afford **52**. Ikeda and coworkers developed a synthetic strategy based on the selective *O*-4-alkylation of **53** by treatment with iodomethane or alkyl bromides in the presence of Ag_2_O and tetra-*n*-butylammonium iodide (TBAI) or in presence of NaH to access C-4 modified zanamivir analogues bearing different aliphatic chains (**55a**–**e**, Scheme 6C) [57]. Compounds **55a**–**e** showed inhibitory activity against the neuraminidase of parainfluenza virus type 1 with the ethylated analogue displaying the greatest efficacy. However, these results were not compared to the inhibitory activity of commercial drugs. Lin and coworkers reported the synthesis of acylguanidine zanamivir derivatives **57** (Scheme 6D) [58]. The synthetic route consisted of the reaction of different acylguanidine derivatives and amine **4** to obtain compounds **56**. Deprotected by treatment with TFA/CH_2_Cl_2_ followed by K_2_CO_3_ afforded derivatives **57** which were evaluated against H1N1 and H3N2 influenza neuraminidases, but showed much lower inhibitory activities in comparison with zanamivir.

**Scheme 7:**
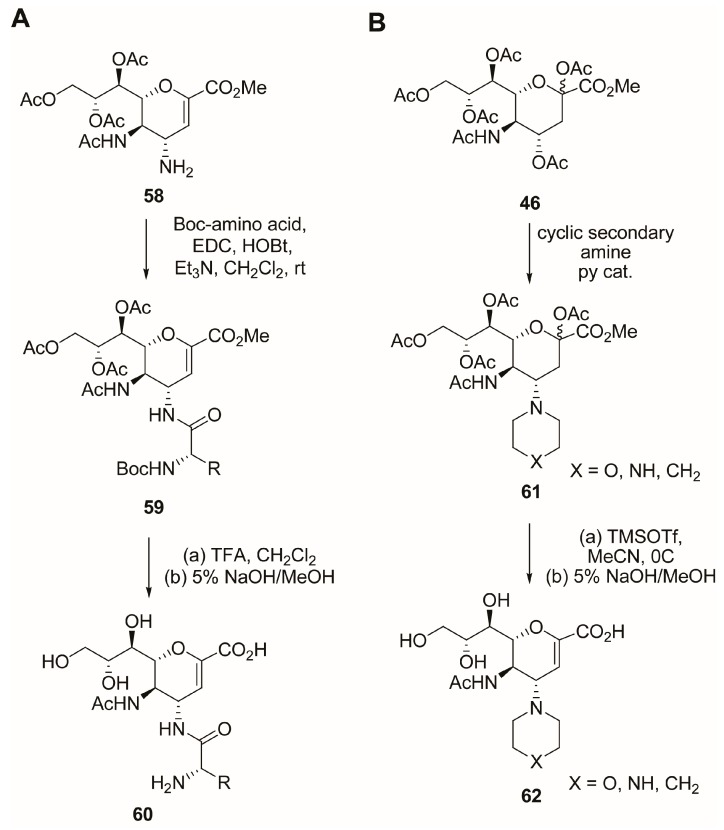
Synthesis of C-4 modified zanamivir analogues (**A**) bearing amino acids (**60**) [59] or (**B**) cyclic secondary amines (**62**) [60].

Liu and coworkers reported the synthesis of a wide range of C-4 modified zanamivir analogues using compounds **46** and **58** as starting materials [59]. The introduction of amino acids was performed by coupling of Boc-protected amino acids with **58** by treatment with 1-hydroxybenzotriazole (HOBt) in presence of *N*,*N*-diisopropylethylamine (DIPEA) and subsequent Ac and Boc deprotection with NaOH/MeOH and TFA/CH_2_Cl_2_, respectively (Scheme 7A). The direct reaction of **46** with substituted isothiocyanates and isocyanates was also performed in a similar manner as reported by Yao and coworkers [44], using **46** instead of **15** as the starting material. The reaction was carried out at room temperature (rt) without addition of any catalyst. Liu and coworkers described the introduction of cyclic secondary amines from acetylated Neu5Ac using pyridine as catalyst (cat.) [60] (Scheme 7B). Acetylation, treatment with TMSOTf, and deprotection using NaOH/MeOH resulted in C-4 substituted zanamivir analogues **62**. Furthermore, Liu and coworkers studied the inhibitory activity of all the synthetized zanamivir analogues [59]. The C-4 derivatization of zanamivir with thiocarbamates, α-amino acids or cyclic secondary amines led to decreased inhibitory activities against both H3N2 and H5N1 influenza virus neuraminidases. The best results were obtained with a zanamivir analogue bearing an l-asparagine moiety which showed 400- and 200-fold lower inhibitory activity towards H3N2 and H5N1 neuraminidases, respectively, than zanamivir.

### 2.4. C-5 Modifications

Von Itztein, Smith and coworkers developed a synthetic procedure for the synthesis of zanamivir derivatives bearing different substituents through substitution of the *N*-acetyl group [61]. The *N*-Boc protected derivative was synthesized by reacting **47** with Boc anhydride followed by deprotection using NaOMe/MeOH and NaOH. The reduction of the azide with triphenylphosphine and guanylation led to the formation of *N*-Boc protected zanamivir which was treated with methyl trifluoroacetate to obtain the corresponding amine as a suitable intermediate for derivatization at C-5. However, none of these C-5 modifications of zanamivir showed enhanced inhibition against influenza A (serotype is not described) and influenza B.

### 2.5. C-6 Modifications

**Scheme 8:**
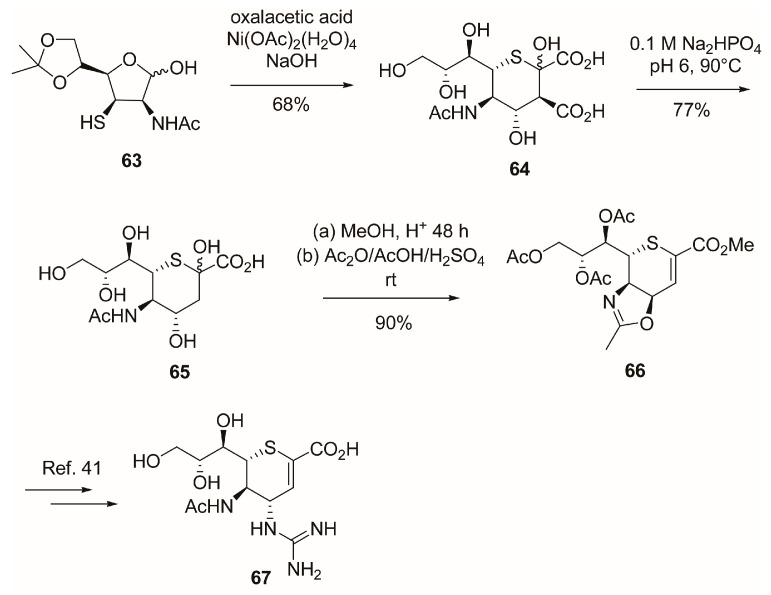
Synthetic strategy for the production of zanamivir C-6 thioether analogues **67** reported by von Itzstein and coworkers [62].

Von Itzstein and coworkers reported the synthesis of the thioether zanamivir derivative **67** (Scheme 8). To achieve this goal, **63** was used as the starting material [62], and was treated with oxalacetic acid in the presence of Ni(OAc)_2_∙(H_2_O)_4_ and NaOH to produce **64**, which was then decarboxylated to give **65**. After protection of the carboxylic acid by treatment with MeOH in the presence of acid, the corresponding ester was acetylated to give the bicyclic compound **66**. The rest of the procedure was carried out in an analogous manner to the reactions described in Scheme 1 [41]. The thioether derivative was found to have inhibitory effects against influenza virus sialidase comparable to its oxy-analogue.

### 2.6. C-7 Modifications (Laninamivir)

**Scheme 9:**
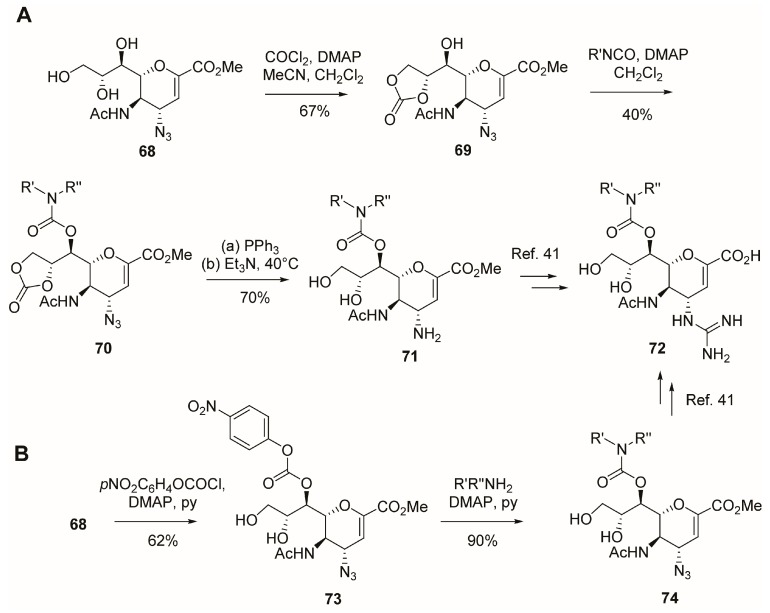
Synthetic routes to C-7 modified zanamivir analogues (**72**) reported by Andrews and coworkers [63]. (**A**) Synthetic approach for the synthesis of mono-substituted carbamates; (**B**) Synthetic approach for the synthesis of carbamates via activation with 4-nitrophenyl chloroformate.

Andrews and coworkers reported the synthesis of C-7 carbamate zanamivir analogues [63]. Two different synthetic methodologies were employed to produce these derivatives. The first (route A, Scheme 9A): began with the protection of compound **68** was by treatment with carbamoyl chloride in presence of 4-dimethylaminopyridine (DMAP) to obtain **69**. **69** was then allowed to react with the appropriate isocyanate, synthesized according to conditions reported by Zbiral and coworkers [64], and DMAP to yield **70**. After reduction of the azido group with triphenylphosphine, the cyclic carbonate was hydrolyzed in aqueous triethylamine at 40 °C to give **71**. The formation of the guanidine moiety was accomplished through the standard technique [41]. In the second approach (route B, Scheme 9B), **68** was treated with 1.2 equivalents of 4-nitrophenyl chloroformate in dry pyridine to yield compound **73**. Treatment of **73** with suitable primary and secondary amines resulted in a panel of C7-carbamates **74**. The rest of the synthesis was carried out as described in route A. C-7 carbamates were obtained with a higher yield (40%–67%) using route B. Furthermore, route B permitted the synthesis of a more diverse range of analogues. Inhibitory activity screening revealed that none of the compounds described were as potent as zanamivir for the inhibition of influenza A and influenza B neuraminidases (the serotype of influenza A was not stated). Klibanov and coworkers have reported the binding of zanamivir to poly(iso-butylene-alt-maleic anhydride) through a C-7 linkage [65], utilizing the method reported by Andrews and coworkers [63]. Although the functionalization with a monofunctional polymer could not improve the inhibitory activity of zanamivir, the bifunctional nature of the polymer allowed the attachment of either zanamivir or Neu5Ac showed in each case an increased inhibitory activity against H3N2 influenza neuraminidase in the order of two magnitudes when compared to zanamivir.

**Scheme 10:**
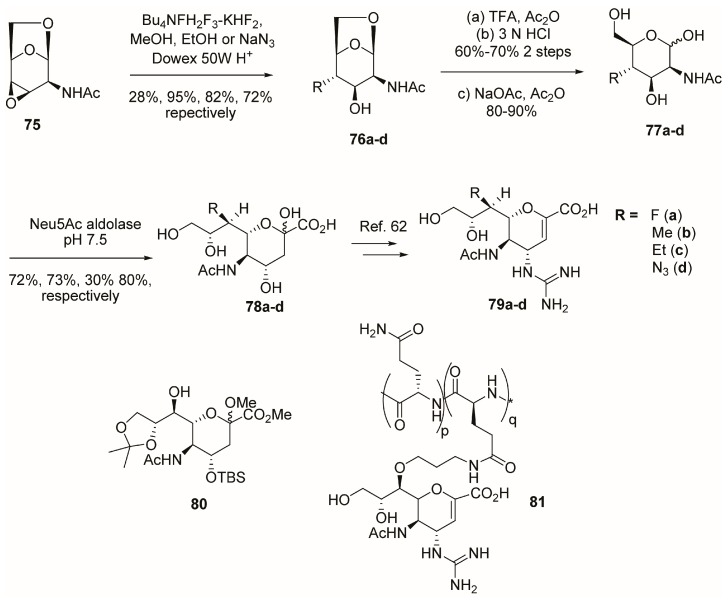
Synthetic route to laninamivir and other zanamivir analogues **79a**–**d** reported by Honda and coworkers [66].

Honda and coworkers developed a chemo-enzymatic route, outlined in Scheme 10, to obtain C-7 substituted zanamivir analogues [66]. Epoxide **75** was subjected to treatment with either Bu_4_NFH_2_F_3_-potassium bifluoride, methanol, ethanol or sodium azide to obtain bicyclic compounds **76a**–**d** with 28%, 95%, 82% and 72% yield, respectively. Acid treatment using TFA followed by 3 N hydrochloric acid allowed the formation of mannose analogues **77a**–**d**. These mannose derivatives **77a**–**d** were then reacted with pyruvate in presence of Neu5Ac aldolase at pH 7.5 to produce the Neu5Ac analogues **78a**–**d**. After protection of the hydroxyl and carboxylic acid groups, the protected sialic acids reacted with sodium azide in the presence of Dowex 50W allowing the incorporation of the azide at C-4 to afford **79a**–**d**. The rest of the synthesis to zanamivir analogues was accomplished according to the previously reported methodology [62]. Compound **80b**, laninamivir, showed an inhibitory activity against influenza B neuraminidase twice as high as that of zanamivir. In 2010, laninamivir was approved for influenza treatment in Japan and is marketed under the name Inavir [67]. Laninamivir is administered by nasal inhalation [67]. A similar synthetic strategy to obtain laninamivir was later reported by Sugai and coworkers [68].

Honda and coworkers also reported direct C-7 alkylation to obtain zanamivir analogues modified with longer side-chains or alcohol, amino, *N*-acetyl, azido or phenyl functionalities. To achieve this, starting material **80** (Scheme 10) was treated with a variety of dialkylsulfates in the presence of NaH in *N*,*N*-dimethylformamide (DMF) to afford the corresponding alkyl ethers in moderate yield. The rest of the procedure to achieve the zanamivir derivatives was carried out according to conditions described above. [62] Neither elongation of the *O*-alkyl chain, nor terminal functionalization of the *O*-alkyl chain with NH_2_, OH, N_3_ and NHAc groups could enhance inhibitory activity against influenza A virus sialidase (serotype is not mentioned).

A direct alkylation methodology was also employed by Honda and coworkers [69] for the synthesis of glutamic acid polymers bearing zanamivir analogues via an alkyl ether spacer linked to the C-7 position. Polyglutamic acid (M. W. 50,000–70,000) was activated with HOBt. Subsequent condensation with the terminal amine linker of zanamivir analogues was afforded the zanamivir-derivatized macromolecules (e.g., **81**, Scheme 10). The efficacy of intranasally administered polymeric sialidase inhibitor **81** was tested in vivo using an infected mouse model on the basis of the survival rate. The inhibitor was administered intranasally once 24 h prior to infection. It was found that compound **81** was a much more effective prophylactic than zanamivir, with a survival rate of 100% among the mice treated with this compound, while none of the mice treated with zanamivir survived.

**Scheme 11:**
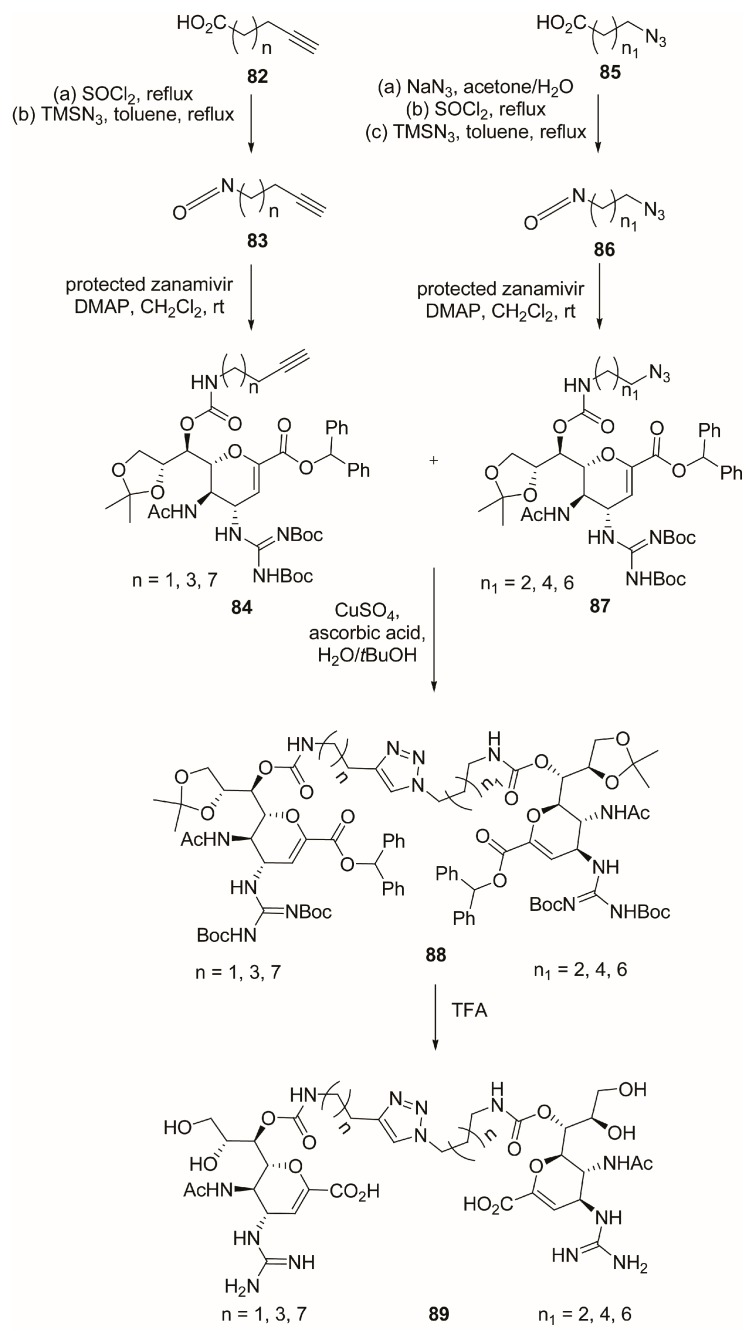
Synthesis of 1,4-triazole linked zanamivir dimers (**89**) reported by Sharpless and coworkers [70].

Sharpless and coworkers reported [70] the synthesis of 1,4-triazole linked zanamivir analogues dimers (**89**) (Scheme 11). Carboxylic acids bearing alkyne functionality (**82**) were treated with thionyl chloride and trimethylsilyl azide to give the corresponding acyl azides which were not isolated but immediately heated to reflux in toluene, inducing Curtius rearrangement to form isocyanates (**83**). The isocyanates thus obtained were reacted with protected zanamivir. Meanwhile, treatment of carboxylic acids **85** with sodium azide in acetone/water gave the corresponding acid azides which were treated with thionyl chloride and trimethylsilyl azide to provide acyl azides. These were then converted via a Curtius rearrangement to the corresponding isocyanates and then allowed to react with protected zanamivir. A 1,-3-dipolar addition reaction between the alkyne-bearing zanamivir derivatives and their azide-bearing counterparts resulted in the formation of dimers **88** which were then deprotected by treatment with TFA. The best conditions for the 1,3-dipolar reaction were found to be CuSO_4_ (0.3 equivalents), ascorbic acid (1.5 equivalents) in a 1:2 H_2_O/*t*BuOH mixture (*v*/*v*) at room temperature. In vitro screening of inhibitory activity revealed that most 1,4-triazole linked zanamivir dimers are significantly more potent inhibitors than zanamivir and oseltamivir against neuraminidase of influenza A (Sydney/5/97, H3N2) and influenza B neuraminidase (Harbin/7/94).

### 2.7. C-9 Modifications

**Scheme 12:**
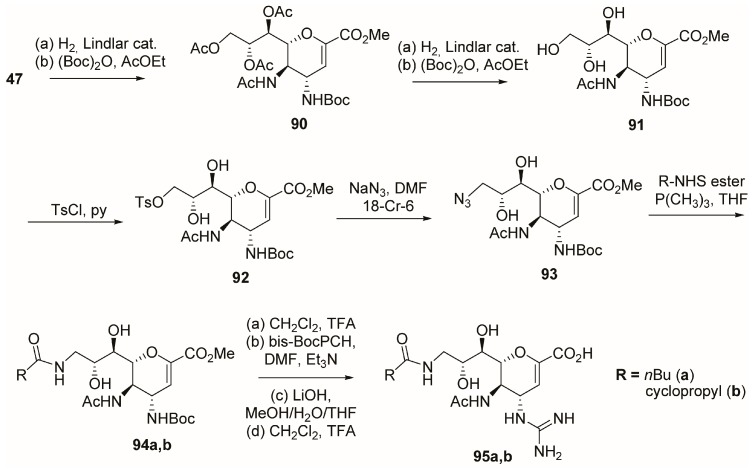
Synthetic strategy to afford C-9 modified zanamivir analogues **95a**–**b** reported by Suzuki, Kiso, Tokiwa and coworkers [71].

Zanamivir analogues bearing 9-cyclopropanecarbonylamino and 9-butanecarbonylamino groups (**95a** and **95b**, respectively) have been developed by Suzuki, Kiso, Tokiwa, and coworkers [71] (Scheme 12). Compound **47** was used as the starting material for their synthetic route. Hydrogenolysis of the azido group of **47** with hydrogen and Lindlar catalyst yielded the corresponding amine derivative which was protected with Boc anhydride resulting in compound **90**. Deprotection of the acetate groups was carried out in NaOMe/MeOH to afford **91**. The C-9 hydroxyl group was selectively activated with *p*-toluenesulfonyl chloride (TsCl) to obtain **92**, which was substituted with azide to obtain **93**. **93** was then subjected to a Staudinger reduction using trimethyl phosphine to generate the intermediate amine which was converted to compounds **94a** and **94b** using the appropriate NHS ester. After removal of protecting groups and guanylation with *N*,*N*′-bis-(tert-butoxycarbonyl)-1*H*-pyrazole-1-carboxamidine (bis-BocPCH), **95a** and **95b** were obtained. These zanamivir analogues were tested against H1N1 and H3N2 influenza virus neuraminidases, however, they displayed lower levels of inhibitory activity in comparison to zanamivir itself.

### 2.8. Other Modifications

**Scheme 13:**
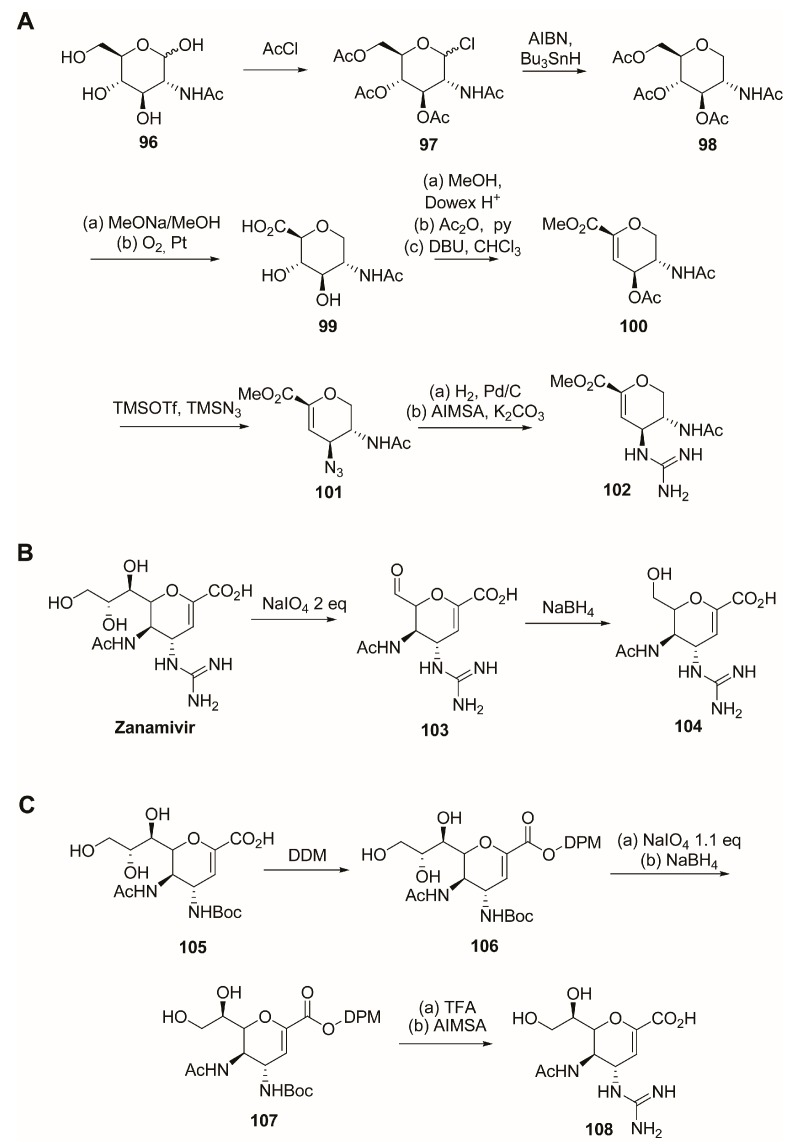
Synthetic strategy reported by Bamford and coworkers [72] to obtain zanamivir analogues wherein the C-6-glycerol side-chain is truncated. (**A**) Synthetic route to zanamivir analogue **102**; (**B**) Synthetic route to zanamivir analogue **104**; (**C**) Synthetic route to zanamivir analogue **108**.

Bamford and coworkers reported the synthesis of zanamivir analogues with truncated C-6-glycerol side-chains (**102**, **104** and **108**) [72]. A zanamivir analogue lacking any side-chain (**102**) was obtained using *N*-acetylglucosamine, GlcNAc (**96**), as the starting material (Scheme 13A). From **96**, the tri-*O*-acetyl-1-chloro derivative **97** was prepared through treatment with acetyl chloride. Azobisisobutyronitrile (AIBN) and Bu_3_SnH were used for the free-radical-initiated dehalogenation to give **98**. After removal of the acetyl protecting groups using NaOMe/MeOH, the primary alcohol was selectively oxidized under oxygen atmosphere in presence of Pt to obtain acid **99**. After esterification of **99** with methanol, the hydroxyl groups of the corresponding ester were protected as acetates using acetic anhydride. An elimination reaction was carried out on the protected compound through treatment with 1,8-diazabicyclo(5.4.0)undec-7-ene (DBU) in CHCl_3_ at reflux to yield **100**, which was reacted with TMSOTf followed by TMSN_3_ to obtain azide **101**. After reduction of the azide under an atmosphere of hydrogen in presence of Pd/C, the resulting amine was guanylated to give zanamivir derivative **102**. The synthesis of the single carbon side-chain analogue **104** was carried out using zanamivir as the starting material. Treatment with sodium periodate (2 equivalents, Scheme 13B) yielded aldehyde **103**, which was directly reduced with sodium borohydride to obtain **104**, after purification by anion-exchange chromatography. The 2 carbon side-chain analogue was obtained from **105** which was treated with diazodiphenylmethane (DDM) to give the DPM ester **106** (Scheme 13C). The oxidation/reduction methodology described for the synthesis of the single carbon side-chain analogue was then employed using 1.1 equivalents of sodium periodate instead of 2 equivalents to afford **107**. Boc and DPM protecting groups were removed using TFA, and subsequent guanylation in presence of aminoiminomethanesulfinic acid (AIMSA) afforded zanamivir analogue **108**. Inhibitory activity screening revealed that compounds, **102**, **104** and **108**, show lower inhibitory activity in comparison with zanamivir against influenza A and influenza B neuraminidases (subtypes are not identified). The highest inhibitory activity was achieved when the two carbon side-chain analogue was used.

**Scheme 14:**
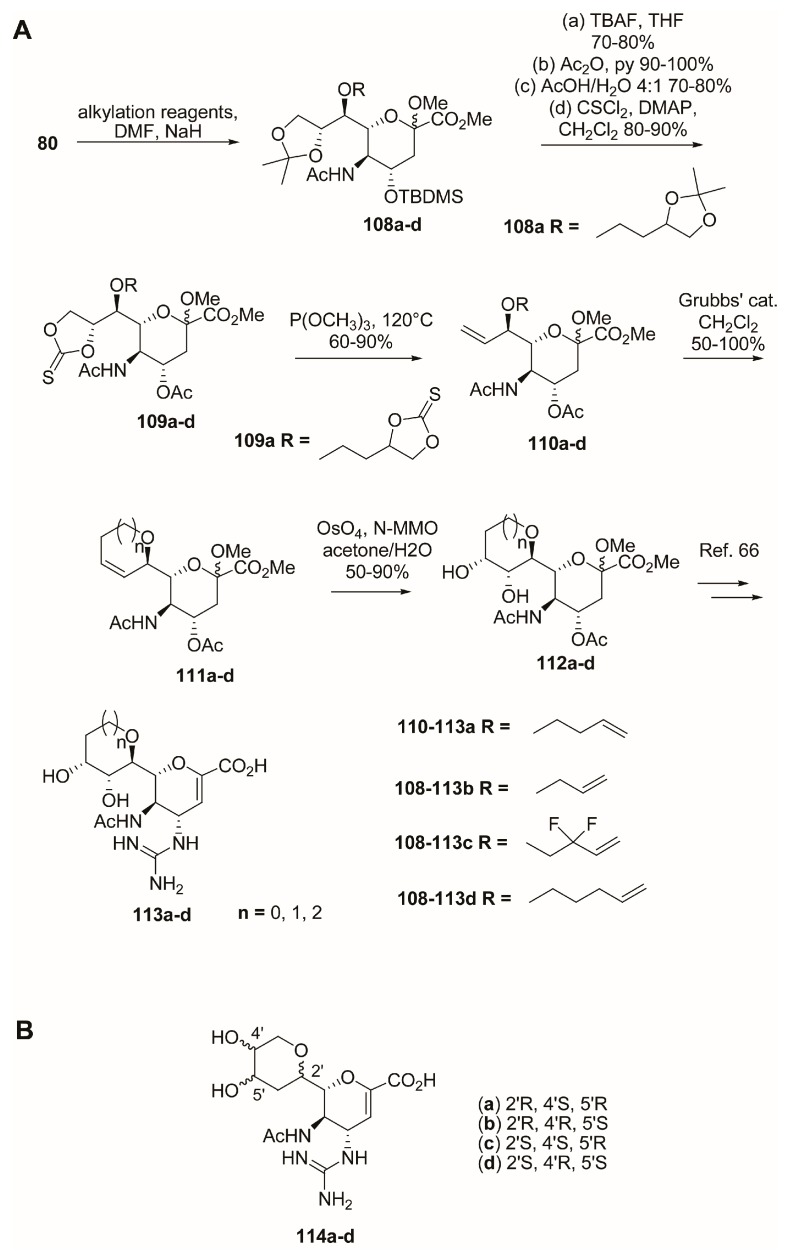
The synthetic route reported by Honda and coworkers [73] to achieve bicyclic ether derivatives of zanamivir. (**A**) Synthetic route to zanamivir analogues **113a**–**d**; (**B**) Zanamivir analogues **114a**–**d**.

Honda and coworkers described a synthetic route to bicyclic ether, namely tetrahydrofuran-2-yl, tetrahydropyran-2-yl and oxepan-2-yl derivatives of zanamivir [73]. The synthesis of the tetrahydrofuran-2-yl, tetrahydropyran-2-yl, and oxepan-2-yl derivatives substituted by diols at the C-3′ and C-4′ positions (**113a**–**d**) was achieved using **80** as the starting material (Scheme 14A). **80** was alkylated with toluene-4-sulfonic acid 2-(2,2-diethyl-(1,3)dioxolan-4-yl)-ethyl ester, allyl iodide, trifluoromethanesulfonic acid 2,2-difluoro-but-3-enyl ester, or 5-iodo-pent-1-ene in the presence of NaH in DMF to give the corresponding compounds **108a**, **108b**, **108c** or **108d**, respectively. After removal of the TBDMS protecting group with TBAF and subsequent protection of the C-4 hydroxyl group with an acetyl group, the acetonide group was deprotected with acetic acid. Compounds **109a**–**d** were then afforded through formation of the thiocarbonates with thiophosgene and DMAP. The thiocarbonates were reduced using methyl phosphite at 120 °C to give compounds **110a**–**d**. A ring-closing metathesis reactions with Grubbs’ catalyst was accomplished to obtain compounds **111a**–**d**. Osmium tetraoxide and *N*-methylmorpholine-*N*-oxide (NMO) were used to selectively oxidize the double bond of **111a**–**d** to provide diols **112a**–**d** as single diastereomers. The diols thus obtained were converted to compounds **113a**–**d** under the same conditions as those previously described in Scheme 10 [66]. Tetrahydropyran-2-yl derivatives substituted by hydroxyl groups at the C-4′ and C-5′ positions (**114a**–**d**, Scheme 14B) were achieved using a similar synthetic strategy. A sialidase inhibitory assay showed that these zanamivir derivatives exhibited inhibition of A/PR/8/34 comparable to that of zanamivir. On the other hand, the movement of the hydroxyl groups from C-3′ and C-4′ (**113a**–**d**) to C-4′ and C-5′ (**114a**–**d**) decreased the inhibitory activity, as did the absence of hydroxyl groups at these positions.

**Scheme 15:**
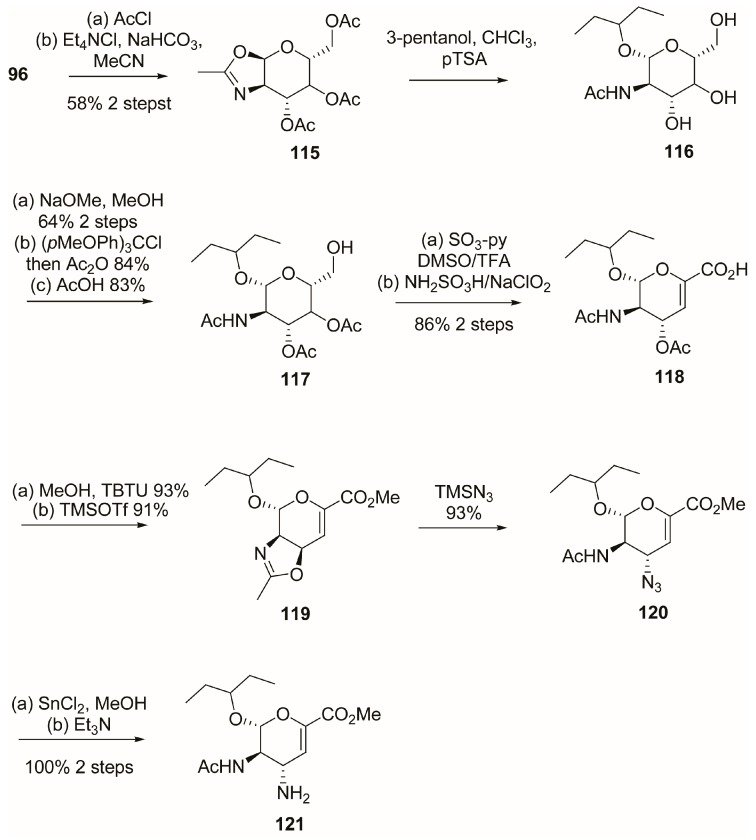
Synthetic strategy reported by Smith and coworkers [74] for the synthesis of zanamivir analogue **121**.

**Figure 3:**
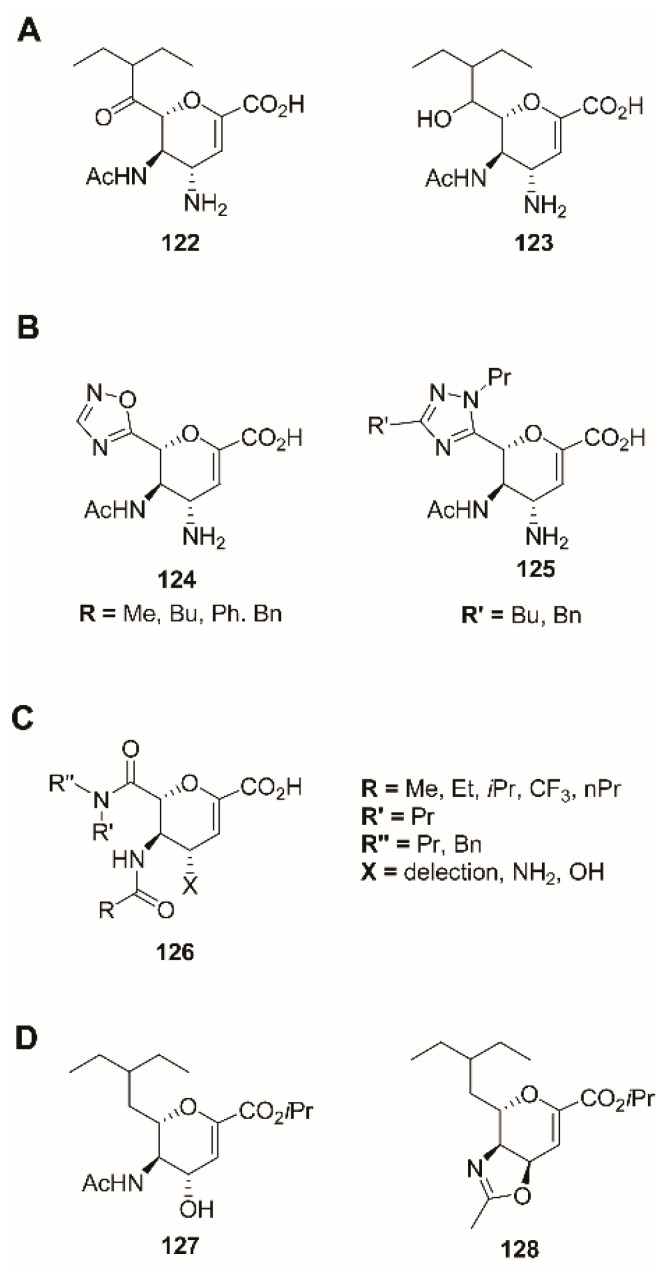
Complex zanamivir analogues. (**A**) Zanamivir analogues **122** and **123** synthetized by Smith and coworkers [74]; (**B**) Zanamivir analogues **124** and **125** synthetized by Smith and coworkers [75]; (**C**) Zanamivir analogue **126** synthetized by Wyatt and coworkers [76]; (**D**) Zanamivir analogues **127** and **128** synthetized by Beau and coworkers [77].

Smith and coworkers explored a different synthetic approach to obtain C-6 ether modified 4-amino zanamivir analogues (**121**) [74] (Scheme 15). Initial chloroacetylation of **96** with acetyl chloride and subsequent cyclization with tetraethylammonium chloride formed the tri-*O*-acetyl oxazoline glycoside **115** which was opened by treatment with 3-pentanol in the presence of *p*-toluene sulfonamide (pTSA) to form **116** exclusively. The secondary alcohol groups were then selectively protected in three simple protecting group manipulations to afford **117**, the unprotected primary alcohol of which was then oxidized with SO_3_-py and NH_2_SO_3_H to afford the α,β-unsaturated acid **118**. This acid was then converted into its methyl ester. Treatment of the ester with 2-(1*H*-benzotriazole-1-yl)-1,1,3,3-tetramethyluronium tetrafluoroborate (TBTU) and TMSOTf produced the oxazoline **119** which was opened with TMSN_3_ to produce azide **120**. Reduction of the azide with SnCl_2_ and hydrolysis of the methyl ester led to zanamivir analogue **121**. Using a similar synthetic route, Smith and coworkers reported the synthesis of a C-6 ketone, 4-amino zanamivir analogue **122** and its reduced derivative **123** [74] (Figure 3A). Smith and coworkers later described [75] the synthesis of oxadiazoles (**124**) and 4-aminozanamivir analogues possessing triazole moieties (**125**, Figure 3B). Both **124** and **125** derivatives showed decreased inhibitory activities with respect to zanamivir. Wyatt and coworkers reported [76] a synthetic approach to C-4 and C-5, 6-carboxamide modified zanamivir analogues (**126**, Figure 3C). In contrast to zanamivir, these analogues were found to be potent inhibitors of influenza A neuraminidase (serotype not specified) when the guanidine group was replaced by amine, hydroxyl or even deleted. While the synthesis of C-5 modified zanamivir analogues was also performed, they showed decreased inhibitory activities in comparison with zanamivir analogues bearing an acetyl group in this position. Inhibitory activities were not compared with zanamivir itself, or with other commercial drugs. On the other hand, Beau and coworkers described [77] a short synthetic route to C-4 zanamivir congeners **127** and **128** with truncated side-chains (Figure 3D) through a Petasis-borono Mannich reaction. No inhibitory activities were reported for derivatives **121**, **122**, **123**, **127** and **128**.

## 3. Oseltamivir

Oseltamivir, commercialized in its phosphate form under the name Tamiflu, was approved in 2002 as an orally administered drug for the treatment of influenza A and B. Oseltamivir is not itself effective against viral neuraminidases, but is rapidly converted by hepatic carboxylases into the potent neuraminidase inhibitor oseltamivir carboxylate. Although Scicinski and coworkers studied several carbocyclic analogues of zanamivir [78], oseltamivir itself was discovered by Bischofberger and coworkers and patented in 1995 [79]. Oseltamivir displays improved inhibitory activity over zanamivir against influenza H2N2, H3N2 and H6N2 neuraminidases [24]. The development of efficient synthetic routes to this compound has been a highly active area of research in the last two decades [80].

### 3.1. Synthesis of Oseltamivir

**Scheme 16:**
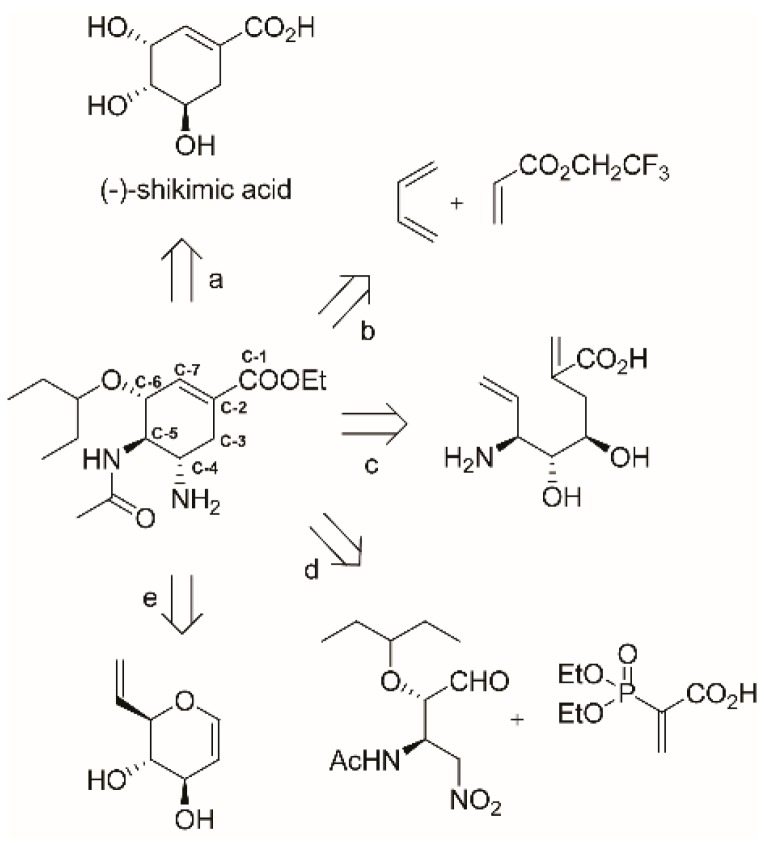
Retrosynthetic analysis of the synthesis routes to oseltamivir. (**a**) synthesis from (−)-shikimic acid or other 6-membered rings; (**b**) through a Diels-Alder reaction with acrylic acid as the dienophile; (**c**) by construction of a cyclohexane ring through an intramolecular metathesis reaction, via a Horner-Wadsworth-Emmons reaction or aldol condensation; (**d**) from nitroalkenes by Curtius rearrangement or (**e**) from d-glucal by Claisen rearrangement.

Several synthetic routes for the synthesis of oseltamivir have been reported and can be broadly divided into five different retrosynthetic strategies: synthesis from (−)-shikimic acid or other 6-membered rings (Scheme 16a), through a Diels-Alder reaction with acrylic acid as the dienophile (Scheme 16b), by construction of a cyclohexane ring through an intramolecular metathesis reaction, via a Horner-Wadsworth-Emmons reaction or aldol condensation (Scheme 16c); from nitroalkenes by Curtius rearrangement (Scheme 16d) or from d-glucal by Claisen rearrangement (Scheme 16e).

**Scheme 17:**
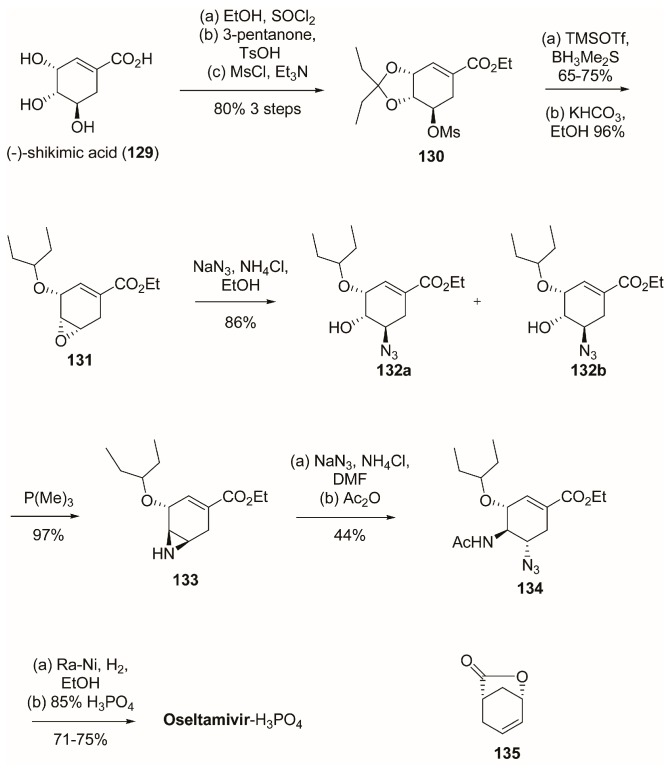
The synthetic approach to (−)-shikimic acid developed by Rohloff and coworkers for the synthesis of oseltamivir [81].

Rohloff and coworkers reported the first synthetic route to oseltamivir starting from the relatively inexpensive (−)-shikimic (Scheme 17) and (−)-quinic acids [81]. This methodology, with minor modifications, has been used for the industrial production of oseltamivir on a multiton scale. The synthesis of acetonide **130** from shikimic acid was accomplished by treatment with TsOH and 3-pentanone, while the free hydroxyl group was protected as a mesylate (Ms). Trimethylsilyl trifluoromethanesulfonate (TMSOTf) and borane-methyl sulfide complex were used for the synthesis of epoxide **131**. The epoxide was opened by azide to obtain compounds **132a** and **132b**. The intramolecular reductive cyclization of **132a** and **132b** was carried out with trimethylphosphine in anhydrous acetonitrile at 35 °C to give aziridine **133** which was opened with sodium azide and ammonium chloride in dimethylformamide to yield azidoacetamide **134** after acetylation of the amine. Compound **134** was then treated with Raney nickel (Ra-Ni) under a hydrogen atmosphere, yielding oseltamivir with a total yield of 35%–40%. Since 1999 several groups have improved upon the original synthesis, increasing the overall yield [82,83,84,85,86,87,88,89]. Other research groups have developed synthetic strategies starting from other 6-membered rings. In two examples of this approach, Hudickly et al. [90] and Kann and coworkers [91] described synthetic routes to oseltamivir using inexpensive ethyl benzoate as the starting material, and Zutter and coworkers accomplished the synthesis of oseltamivir starting from 2,6-dimethoxyphenol [92]. Raghavan et al. and Rohloff et al. reported synthetic approaches to oseltamivir using 3-cyclohexene carboxylic acid as the starting material [81,93], whereas a *cis*-1,2-dihydrodiol bromoarene was employed in the synthetic route reported by Fang and coworkers [94]. Trost and Zhang [95] used a bicyclic lactone **135** (Scheme 17) which could be asymmetrically alkylated through the use of a catalytic palladium complex to provide a chiral intermediate product for the synthesis of oseltamivir.

**Scheme 18:**
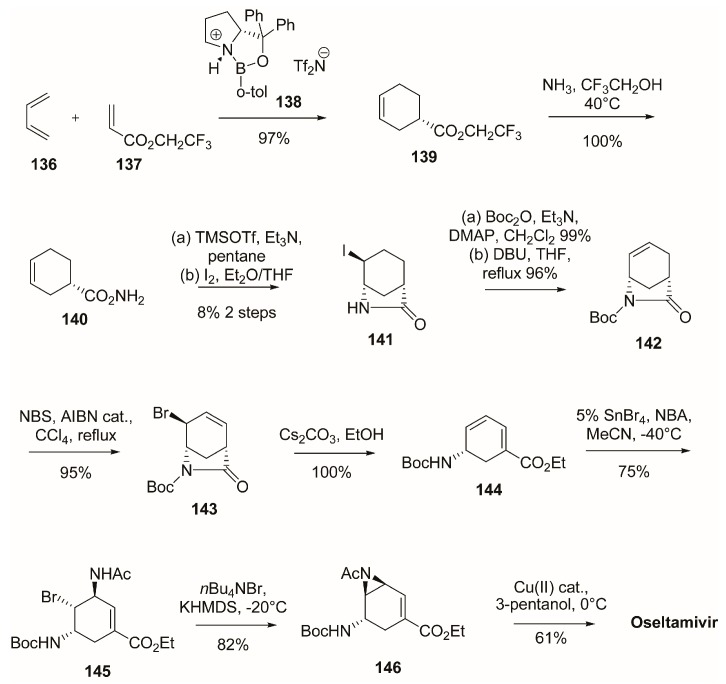
Synthetic strategy for the synthesis of oseltamivir developed by Corey and coworkers [96].

Corey and coworkers developed a synthetic route based on a Diels-Alder reaction. The procedure began with a [4 + 2] cycloaddition between 1,3-butadiene (**136**) and trifluoroethyl acrylate (**137**) using an *S*-proline-derived Lewis-acid catalyst (**138**) obtaining **139** [96] (Scheme 18). Ammonolysis of the ester group of **139** was accomplished by treatment with ammonia in presence of TFA. Compound **141** was then produced by reaction of **140** with iodine. After protection of the amine with Boc, a dehydroiodination reaction was carried out with DBU to give **142**, which was allylically brominated using *N*-bromosuccinimide to generate **143**. Treatment of **143** with cesium carbonate in ethanol yielded compound **144**, which was subjected to a SnBr_4_-catalyzed bromoacetamidation reaction using *N*-bromoacetamide (NBA) in acetonitrile at −40 °C to obtain **145**. The construction of the aziridine was performed using tetra-*n*-butylammonium hexamethyldisilazane and provided bicyclic product **146**. After treatment with cupric triflate and 3-pentanol at 0 °C, and removal of the Boc protecting group, oseltamivir was afforded. Other Diels-Alder-based approaches to this molecule include that of Fukuyama and coworkers, who subjected pyridine to a Diels-Alder reaction with acrylic acid derivatives using McMillans catalyst [97], while Wu and coworkers have reported a synthetic strategy which starts from the Diels-Alder cycloaddition between *N*-Boc pyrrol and ethyl 3-bromopropyolate [98], and Shibasaki and coworkers have reported the synthesis of oseltamivir starting from fumaryl chloride and 1-(*t*-butyldimethylsolix)-1,3-butadiene [99,100].

**Scheme 19:**
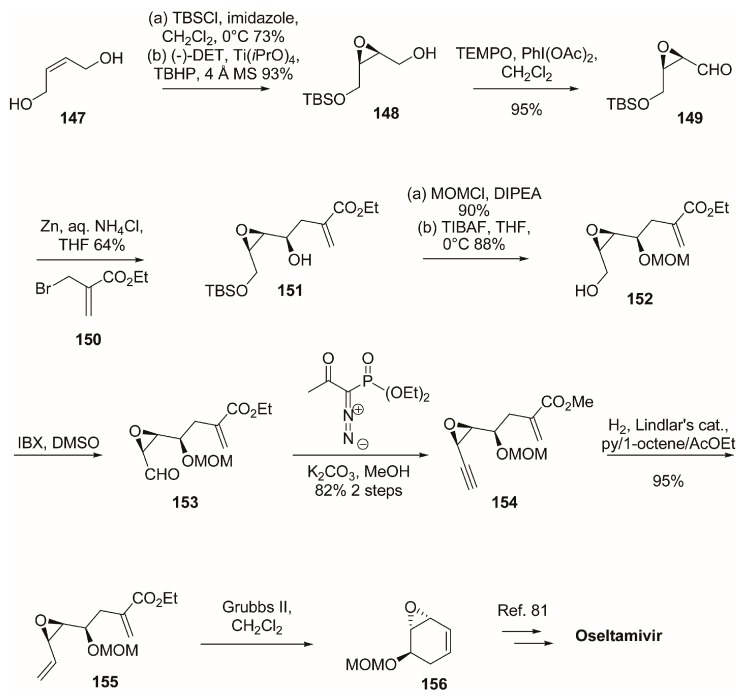
Synthetic route from *cis*-1,4-butenediol **147** proposed by Sudalai and coworkers [101] for the synthesis of oseltamivir based on the formation of the ring by a metathesis reaction.

Another approach to the synthesis of oseltamivir is based on a ring formation by a metatesis reaction. This strategy was applied by Sudalai and coworkers using *cis*-1,4-butene diol (**147**) as the starting material (Scheme 19) [101]. **147** was monosilylated with TBSCl and then treated with tert-butyl hydroperoxide (TBHP) in presence of (−)-DET to give the epoxide **148**. 2,2,6,6-tetramethylpiperidinyloxy (TEMPO) was used for the selective oxidation of the free hydroxyl group to yield aldehyde **149**, which was subjected to allylation with ethyl 2-(bromomethyl)acrylate (**150**) and zinc to obtain **151**. The hydroxyl group of **151** was then protected with MOMCl and the TBS group removed to yield **152**, which was oxidized with 2-iodoxybenzoic acid (IBX) to obtain the aldehyde **153**. A Seyferth-Gilbert homologation was performed to achieve **154** and then the triple bond was reduced to a double bond under a hydrogen atmosphere in the presence of Lindlar catalyst. The cyclohexene core **156** was then constructed via a metathesis reaction using Grubbs’ II catalyst. Finally, oseltamivir was achieved using similar conditions than those reported by Rohloff and coworkers [81] and Nie and coworkers [84]. A similar synthetic approach was reported by Kang and Oh [102] using *cis*-2,3-bis(hydroxymethyl)aziridine instead of the epoxide derivative **148**. Yao and Cong developed a synthetic strategy starting from l-serine [103] whereas protected (*S*)-glutamic acid was used in the synthesis of oseltamivir proposed by Saicic and coworkers [104].

**Scheme 20:**
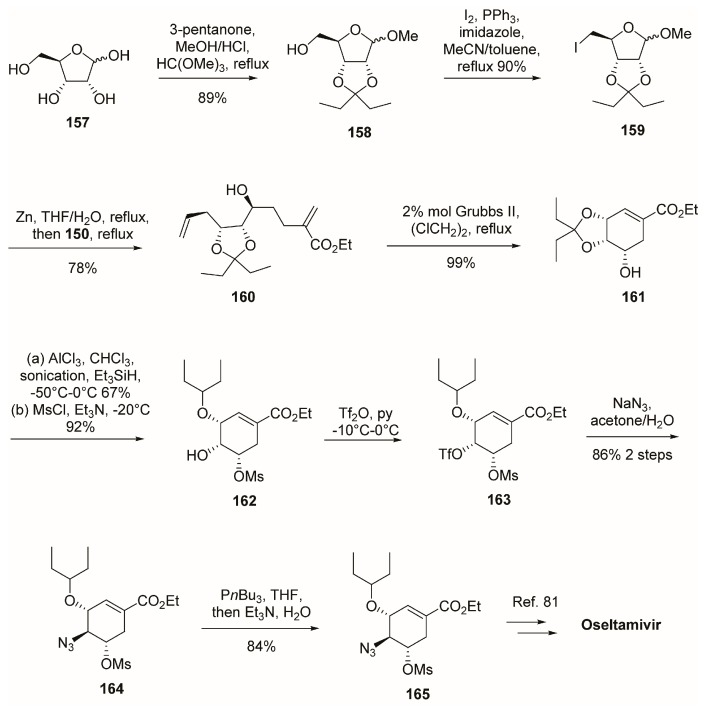
Synthetic route from d-ribose (**157**) proposed by Chai and coworkers [105] for the synthesis of oseltamivir, the key step of which consists of the formation of the ring by a metathesis reaction.

Chai and coworkers reported a synthetic route to oseltamivir from d-ribose (**157**) the key step of which consists of an intramolecular metathesis reaction to afford the six-member ring (Scheme 20) [105]. After protection of d-ribose with methanol and 3-pentanone, compound **158** was treated with iodine in the presence of imidazole and PPh_3_ to afford **159**. Zn-mediated elimination-allylation of **159** provided **160**, which was subjected to a metathesis reaction using a Grubbs’ II catalyst to afford **161**. After opening of the acetonide ring with aluminium chloride, the hydroxyl group linked to C-4 was selectively mesylated to give **162**. Treatment of **162** with trifluoromethanesulfonic anhydride in the presence of pyridine allowed the formation of **163**. After the introduction of an azide at C-5, aziridine **165** was obtained by reduction of the azide to the corresponding amine via Staudinger reaction followed by trimethylamine-mediated cyclization. Finally, oseltamivir was afforded according to conditions reported by Rohloff and coworkers [81]. A similar synthetic strategy from ribose was also reported by Kongkathip and coworkers shortly after [106]. Recently, Kongkathip and coworkers have again described a synthetic route with minor modifications using d-glucose as the starting material [107].

**Scheme 21:**
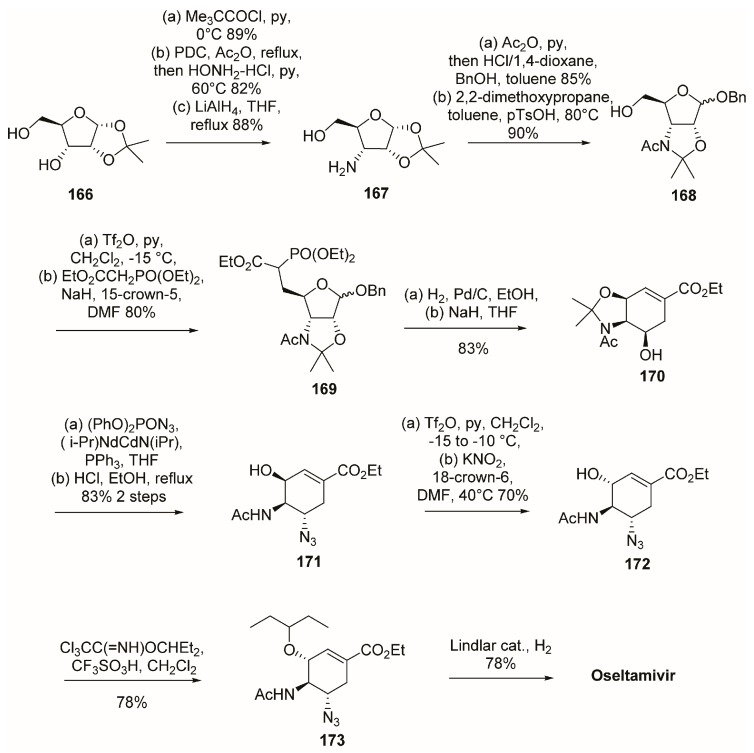
Synthetic route proposed by Shie and coworkers [108] for the synthesis of oseltamivir using mannitol as the starting material and constructing the ring via a Horner-Wadsworth-Emmons reaction.

Shie and coworkers developed a synthetic approach based on the construction of the carbocyclic ring via the Horner-Wadsworth–Emmons reaction [108] (Scheme 21). 1,2-Di-*O*-isopropylidene-α-d-xylofuranose (**166**) was treated with NH_2_OH∙HCl and pyridinium dichromate (PDC) followed by LiAlH_4_ to obtain **167**, which was protected with acetic anhydride, 2,2′-dimethoxypropane and benzyl alcohol.

The primary hydroxyl group of **168** was then replaced by ethoxycarbonylmethanephosphonic acid diethyl ester in using NaH and a 15-crown-5 catalyst to give **169**. An intramolecular Horner-Wadsworth-Emmons reaction was then carried out to yield the cyclohexene carboxylate **170**. After introduction of the azido group in C-4, the acetal protecting group was removed and C-6 was epimerized to afford **172**. **172** was reacted with Cl_3_C(=NH)OCHEt_2_ and the compound **173** azide was reduced under a hydrogen atmosphere in presence of Lindlar catalyst to obtain oseltamivir. Later, Kongkathip and coworkers [109] and Fang and coworkers [110] succeeded in performing the ring closure by an intramolecular Horner-Wadsworth-Emmons reaction using mannose and *N*-acetylglucosamine as starting materials, respectively.

**Scheme 22:**
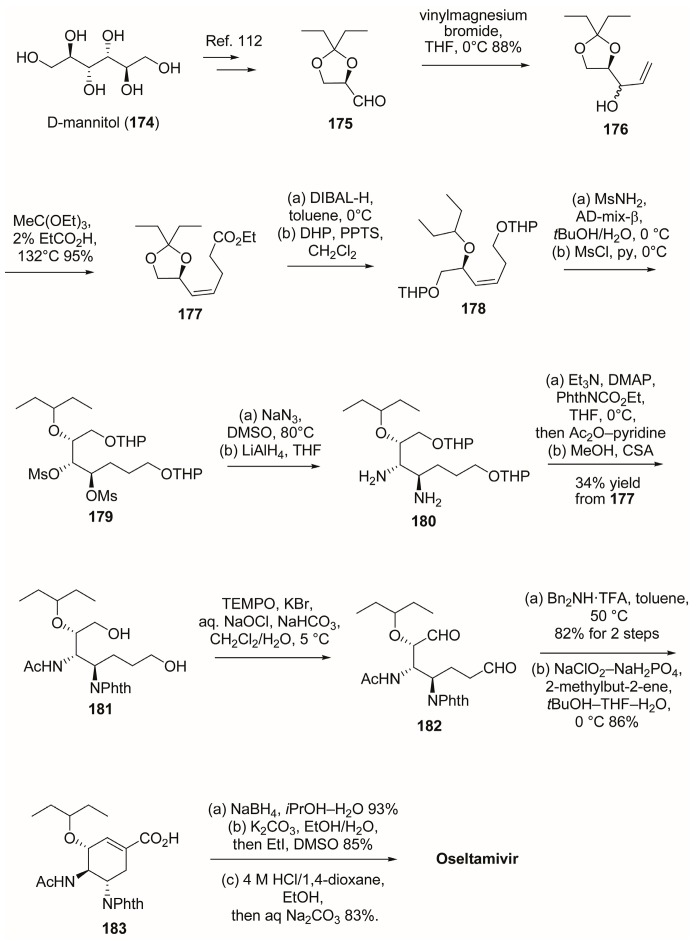
Synthetic route from mannitol (**174**) to oseltamivir reported by Mandai and coworkers [111] performing the construction of the ring via aldol condensation.

Mandai and coworkers described a synthetic route based on the same retrosynthetic analysis but in this case performing the construction of the ring via an aldol condensation [111] (Scheme 22). Mannitol (**174**) was used as the starting material, and transformed into the aldehyde form **175** using periodate-based oxidation [112]. **175** was treated with vinylmagnesium bromide to give **176**, which was subjected to Claisen rearrangement to produce ester **177**. The ester was reduced to a hydroxyl group with DIBAL, which was then protected with a 2tetrahydropyranyl group (THP) to give **178**. AD-mix-β was used to dihydroxylate **178** followed by mesylation of the hydroxyl groups to obtain **179**. The mesylated alcohols were substituted for azides, which were reduced to amines by treatment with lithium aluminium hydride (LiAlH_4_) to give **180**. Amines were protected regioselectively by treatment with *N*-ethoxycarbonylphthalimide (PhthNCO_2_Et) and acetic anhydride to provide **181** after deprotection of the THP groups. Hydroxyl groups were then oxidized to aldehydes using TEMPO. Ring-closure was performed via an aldol condensation in presence of Bn_2_NH·TFA to afford **183**. Finally, deprotection led to oseltamivir. Later, a similar synthetic approach was reported by the same research group using methionine as the starting material [113]. The Ko research team simplified the mannitol-based synthesis by protecting the carboxylic acid of **117** as a lactone [114]. A Dieckmann condensation was used by Shibasaki and coworkers for the construction of the oseltamivir ring intermediate **144** [115], which was also reported by Corey and coworkers [96].

**Scheme 23:**
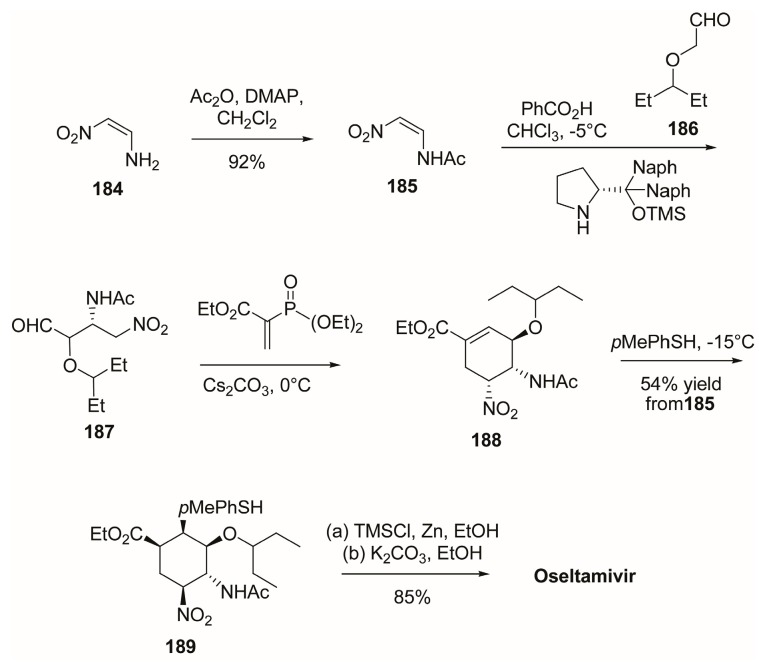
Synthetic route to oseltamivir based on ring construction via a Curtius rearrangement reported by Ma and coworkers [116].

**Scheme 24:**
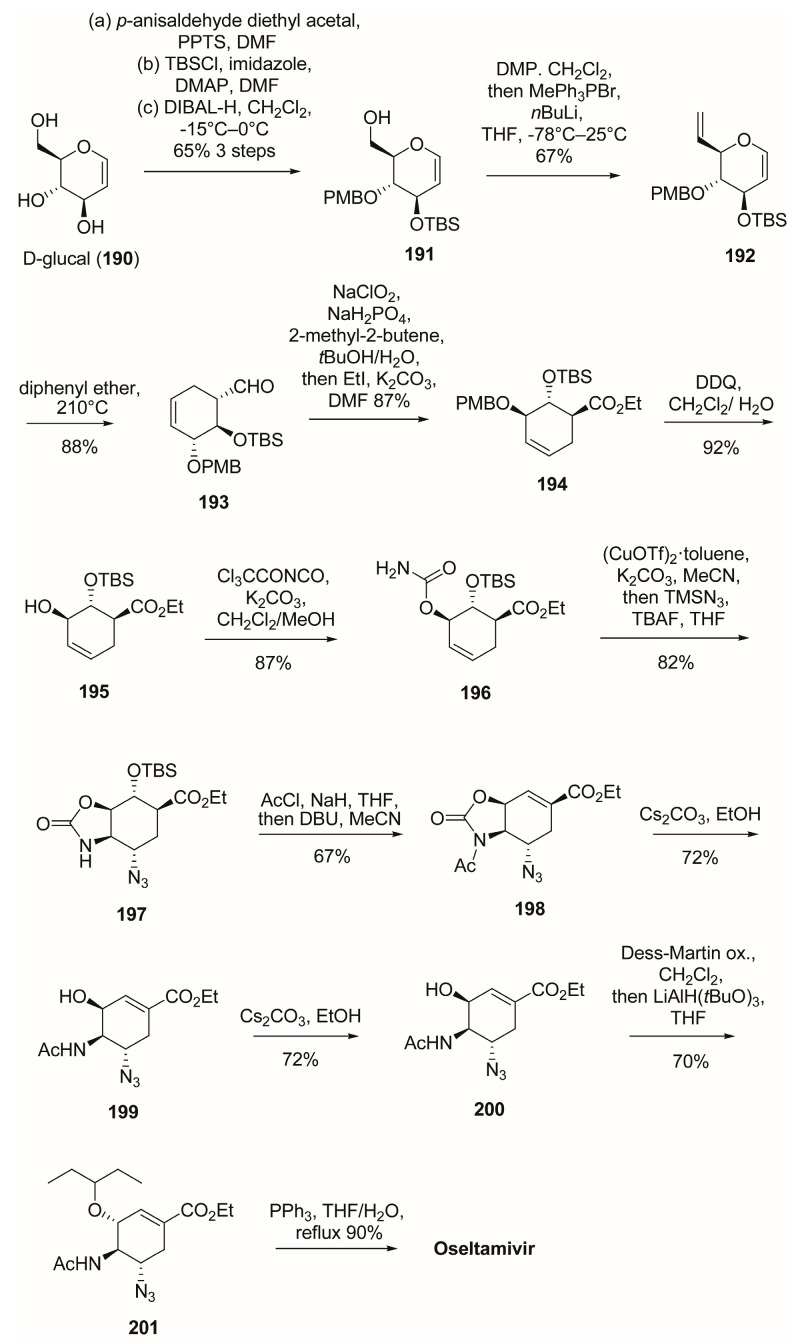
Synthetic route to oseltamivir by Liu and coworkers [121]. The oseltamivir ring was afforded by a Claisen rearrangement.

Ma and coworkers reported a synthetic methodology to obtain oseltamivir with ring construction via Curtius rearrangement [116] (Scheme 23). (*Z*)-2-Nitroethenamine **184** was treated with acetic anhydride and DMAP yielding the enamide **185**, which was subjected to Michael addition with 2-(pentan-3-yloxy)acetaldehyde (**186**) using a proline derivative as a catalyst. Curtius rearrangement was carried out by addition of vinylphosphonate and Cs_2_CO_3_ to give **188**, which was directly treated with *p*-toluenethiol to provide the corresponding ester **189**. **189** was then transformed into oseltamivir after reduction with Zn and K_2_CO_3_ treatment. Later, Šebesta and coworkers [117], Hayashi and coworkers [118,119] and Lu and coworkers [120] reported similar synthetic strategies to obtain oseltamivir using Curtius rearrangements as key steps for the construction of ring. It is worth mentioning that Hayashi and coworkers performed the synthesis to oseltamivir in a one-pot synthesis [119]. Liu and coworkers explored a synthetic approach using a Claisen rearrangement for the construction of the oseltamivir ring using d-glucal (**190**) as the starting material [121] (Scheme 24). It was reported that **190** was synthetized from glucose although reaction conditions of this transformation are not mentioned. Fully protected d-glucal was achieved by formation of a 4,6-benzylidene acetal in presence of pyridinium *p*-toluenesulfonate (PPTS) and silylation of the 3-hydroxyl group, followed by treatment with diisobutylaluminium hydride (DIBAL-H) to free the primary alcohol **191**. The primary hydroxyl group of **191** was oxidized to the aldehyde by using Dess-Martin periodinane and then subjected to Wittig methylenation to provide terminal olefin **192**. The Claisen rearrangement reaction was performed at 210 °C in diphenyl ether to yield **193**. The oxidation of **193** to ethyl ester **194** was carried out by using NaClO_2_/NaH_2_PO_4_ in the presence of 2-methyl-2-butene, followed by esterification with ethyl iodide. 2,3-Dichloro-5,6-dicyanobenzoquinone (DDQ) was used to selectively remove the PMB protecting group to provide **195**. Then, transformation into compound **196** was carried out with trichloroacetyl isocyanate and potassium carbonate. **196** was treated with (CuOTf)_2_-toluene and TMSN_3_ to give **197**. Treatment of **197** with DBU followed by the addition of Cs_2_CO_3_ provided compound **199** which was subjected to treatment with Dess-Martin periodinane and then LiAlH(O*t*Bu)_3_ to promote inversion of configuration at C-3. **201** was generated by treatment with MsCl/Et_3_N followed by 3-pentanol/BF_3_·Et_2_O. Finally, oseltamivir was obtained after reduction of the azido group with PPh_3_ in tetrahydrofuran (THF)/H_2_O.

### 3.2. C-1 Modifications

**Figure 4:**
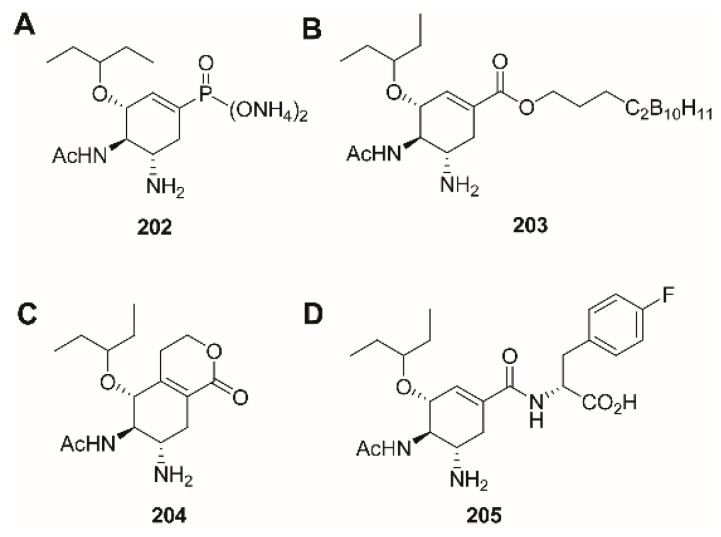
C-1 modified oseltamivir analogues. (**A**) Tamiphosphor (**202**) [108]; (**B**) Oseltamivir bearing a boron cluster reported by Lesnikowski and coworkers [124] (**203**); (**C**) A bicyclic derivative of oseltamivir synthesized by Kanai and Saito [125] (**204**); (**D**) A 4-*F*-phenylalanine (*R*) derived oseltamivir analogue (**205**).

Oseltamivir phosphonate (**202**, Figure 4A), also called tamiphosphor, was synthesized by Shie and coworkers using the same synthetic strategy reported for the synthesis of oseltamivir via intramolecular Horner-Wadsworth-Emmons reaction [108], with the sole difference being that the primary alcohol is substituted with CH_2_(PO(OEt)_2_)_2_ rather than(EtO_2_CCH_2_PO(OEt)_2_), resulting in a phosphonate ester in place of the carboxylate ester. Inhibitory activity screening revealed that the phosphonate analogue is a more potent inhibitor against H1N1 and H5N1 neuraminidases than oseltamivir [108]. Gunasekera [122] and Streicher [123] have both reported the synthesis of tamiphosphor from oseltamivir. Lesnikowski and coworkers reported a synthetic approach to achieve an oseltamivir derivative bearing a boron cluster on C-1 (**203**) [124] (Figure 4B). After the ester hydrolysis of compound **134**, the acid was treated with 1-(3hydroxypropyl)-1,12-dicarba-closo-dodecaborane in the presence of DCC, and the resulting azide reduced with PPh_3_ to provide the desired derivative **203**. Kanai and Saito reported the synthesis of a bicyclic oseltamivir analogue **204** [125] (Figure 4C), which was achieved after functionalization of the C-7-H bond with a Ru catalyst and the subsequent addition of olefins. Stankova and coworkers explored the synthesis of oseltamivir esters of amino acids 4-*F*-phenylalanine (*R*,*S*) and glycine [126]. The resulting oseltamivir derivative with 4-*F*-phenylalanine (*R*) (**205**, Figure 4D) could successfully inhibit the influenza virus in a cell based assay.

### 3.3. C-4 Modifications

**Scheme 25:**
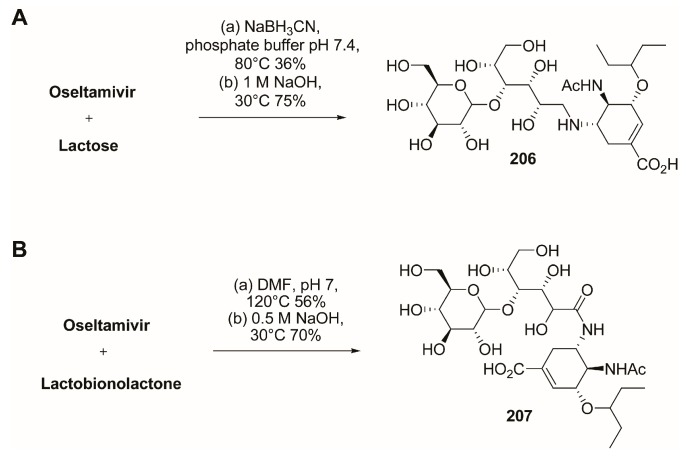
The synthetic approach described by Lederkremer and coworkers to access oseltamivir C-4 lactose analogues [127]. (**A**) Synthetic route to oseltamivir analogue **206**. (**B**) Synthetic route to oseltamivir analogue **207**.

Lederkremer and coworkers described the enzymatic synthesis of oseltamivir C-4 lactose analogues [127]. Two different approaches were used to link the amino group of oseltamivir to lactose and lactobionolactone. The linkage with lactose was performed by reductive amination of its reducing end with oseltamivir in the presence of NaBH_3_CN to provide **206** (Scheme 25A). The amide formation between the carboxyl group of lactobionolactone and the amino group of oseltamivir was performed at 120 °C (pH 7) to yield **207** (Scheme 25B). The *trans*-sialidase of the protozoan parasite *Trypanosoma cruzi*, which allows the enzymatic addition of α(2,3)-linked sialyl residues to the terminal d-galactopyranosyl units of mucins, was used to study the inhibitory activity of the oseltamivir analogues. Both **206** and **207** demonstrated to be stronger inhibitors than oseltamivir against *Trypanosoma cruzi* neuraminidase, while the inhibitory activities of **206** and **207** were inferior to those shown by lactitol and lactobionolactone.

**Scheme 26:**
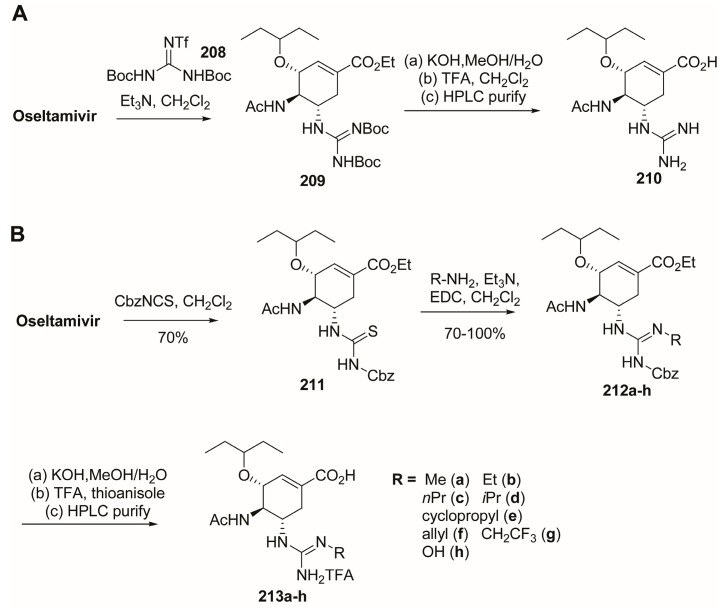
Synthetic routes to oseltamivir analogues bearing a guanidine group linked to C-4. (**A**) Synthetic approach to C-4 guanidine (**210**); (**B**) *N*-substituted guanidine oseltamivir analogues (**213a**–**h**) starting from oseltamivir reported by Martin and coworkers [129].

Chochkova and coworkers reported a synthetic approach to obtain oseltamivir amino acids conjugates using Ac-Cys-OH, Fmoc-Tyr(*t*Bu)-OH and Boc-His(DNP)-OH as building blocks [128]. The C-termini of these compounds were amidated with the amine of oseltamivir using (1-ethyl-3-(3-dimethylaminopropyl)carbodiimide (EDC)/HOBt. Martin and coworkers reported an easy synthetic approach to C-4 guanidine (**210**, Scheme 26A) and *N*-substituted guanidine oseltamivir analogues (**213a**–**h**, Scheme 26B) starting from oseltamivir in a similar approach [129]. The unsubstituted oseltamivir analogue **210** was obtained after reaction of oseltamivir with **208** and the subsequent deprotection of the guanidine and carboxylic groups. For the synthesis of **213a**–**h**, oseltamivir was treated with *N*-benzyloxycarbonyl isothiocyanate (CbzNCS) to yield thiourea **211**. The reaction between **211** and different amines and subsequent deprotection of the guanidine and carboxylic acid groups provided *N*-substituted guanidine oseltamivir analogues **213a**–**h**. **210** was shown to be capable of enhanced the inhibitory activity against H1N1 (A/California/04/2009), H1N1 mutant H274Y (A/California/04/2009), H5N1 (A/Anhui/1/2005) and H5N1 mutant H274Y (A/Anhui/1/2005). This result mirrors the effect of the guanidine modification observed in zanamivir [3,39,40]. While *N*-substituted guanidine oseltamivir analogues **213a** and **213h** showed enhanced inhibitory activity in comparison with oseltamivir against the above mentioned influenza virus strains, they showed less inhibitory activity than compound **210**.

### 3.4. C-5 Modifications

**Scheme 27:**
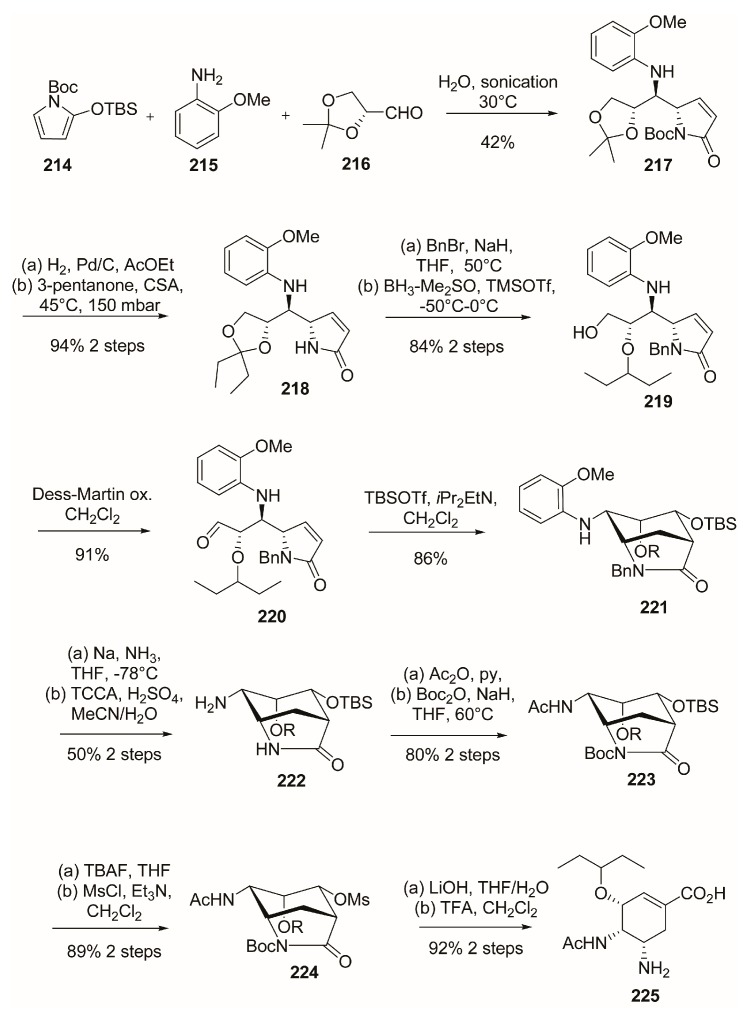
The synthetic route to 5-epi-oseltamivir (**225**) reported by Zanardi and coworkers [130].

Zanardi and coworkers reported a synthetic strategy for the synthesis of 5-epi-oseltamivir **225** [130] (Scheme 27). Pyrrole **214**, d-mannitol-derived glyceraldehyde **215** and *O*-anisidine **216** were used for the production of compound **217** through a Mukaiyama-Mannich reaction performed at 30 °C in water. **217** was subjected to catalytic hydrogenolysis over Pd/C, and the resulting compound was protected by treatment with 3-pentanone and camphorsulfonic acid (CSA) to provide **218**. After protection of the amide with a benzyl group, ring-opening of the ketal was achieved using BH_3_∙Me_2_SO/TMSOTf in THF. The primary alcohol of **219** was oxidized by treatment with Dess-Martin periodinane to obtain **220**, which was subjected to an intramolecular aldol cyclization in the presence of TBSOTf/*i*Pr_2_EtN to produce **221**. After removal of the amine protecting groups by treatment with sodium in ammonia and with trichloroisocyanuric acid (TCCA) and subsequent protection as acetate and Boc, fluoride-promoted *O*-desilylation and mesylation transformed **223** into lactam **224**. Finally, treatment with lithium hydroxide led to a monocyclic carboxylate, and removal of the Boc protecting group and elimination gave **225**. This compound showed a much lower inhibitory activity against H1N1 and H3N2 influenza neuraminidases than oseltamivir. De-Eknamkul and coworkers developed a synthetic approach to 5-amino derivatives from quinic acid [131], following the synthetic strategy described by Rolhoff and coworkers [81]. Azide **134** was acylated with acrylic acid or crotonic acid and the azido group was reduced to amino group. These compounds showed similar inhibitory activities compared to oseltamivir.

### 3.5. C-6 Modifications

Šebesta and coworkers described the synthesis of oseltamivir bearing a benzyloxy group or a *p*-methoxybenzyloxy group at the C-6 position [117]. The synthetic approach used was similar to that reported by Ma and coworkers, i.e., via a Curtius rearrangement but using the benzyloxy and *p*-methoxybenzyloxy derivatives of aldehyde **186** [116].

## 4. Peramivir

Peramivir, also known by its trade names Rapivab, Rapiacta or Peramiflu, is the latest commercialized drug for the treatment of influenza. Peramivir is administered intravenously [21]. It was developed by structure-activity relationship (SAR) studies of oseltamivir, which led to modifications including contraction of the 6-membered ring to a 5-membered ring [132]. Inhibitory studies have revealed that peramivir showes higher inhibition towards H1N1 influenza neuraminidase in comparison to zanamivir and oseltamivir [133,134,135,136]. As this compound was only recently approved in the USA (2014) and in Japan and South Korea (2015), few synthetic routes to peramivir or its derivatives have been reported to date.

### 4.1. Synthesis of Peramivir

**Scheme 28:**
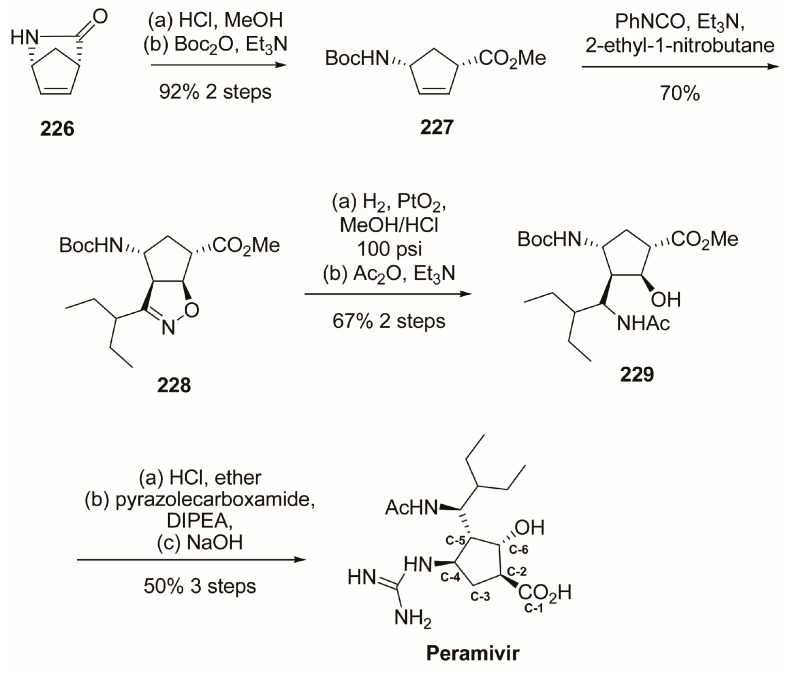
The synthetic route to peramivir using lactam **226** as the starting material reported by Babu and coworkers [132].

All reported synthetic routes are based on the same retrosynthetic approach, using (−)-(1*R*,4*S*)-2-azabicyclo(2.2.1)hept-5-en-3-one (**226**) or derivative **232** as precursors. Babu and coworkers described the first synthetic route to peramivir (Scheme 28) [132]. The opening of lactam ring **226** was achieved through hydrochloric acid treatment. After protection of the amine with Boc, **227** was subjected to a [3 + 2] cycloaddition with 2-ethyl-1-nitrobutane in presence of phenyl isocyanate to obtain **228**. PtO_2_ catalyzed hydrogenolysis and subsequent protection using acetic anhydride yielded compound **229**. After removal of Boc, the corresponding amine was further reacted with pyrazolecarboxamide and then hydrolysed in presence of NaOH to give peramivir with a 21% total yield. Later, Jia and coworkers improved this synthetic route, increasing the overall yield to 34% [137].

**Scheme 29:**
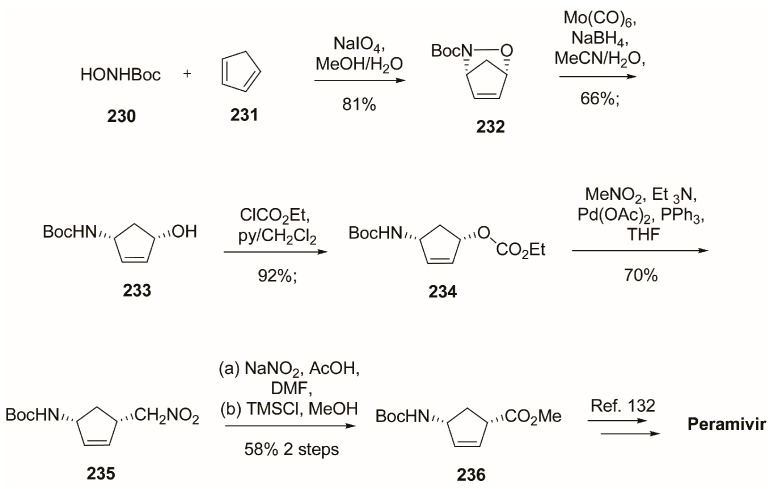
Synthetic route to peramivir using a lactam (**232**) precursor, as reported by Miller and Mineno [138].

Miller and Mineno developed a synthetic approach starting from Boc-protected hydroxylamine (**230**) which was subjected to a Diels-Alder reaction with cyclopentadiene (**231**) to give **232** (Scheme 29) [138]. After ring opening of lactam **232** via Mo(CO)_6_ treatment, the resulting compound **233** was reacted with ethyl chloroformate to yield **234**. This was then treated with MeNO_2_ and a catalytic quantity of Pd(0) to obtain nitro compound **235**, which was transformed into the carboxylic acid by employing a mixture of NaNO_2_ and AcOH in DMF, followed by treatment with a catalytic amount of TMSCl in MeOH to give compound **236**. The remaining steps were carried out in a similar manner to that described by Babu and coworkers to yield peramivir [132].

### 4.2. C-4 Modifications

Wulff and coworkers carried out a study on the inhibitory activity of de-guanidinylated peramivir analogue [139], with the results suggesting that the lack of the guanidine group in the peramivir structure had no effect on the inhibitory activity against H1N1 neuraminidases. The synthesis of the de-guanidinylated analogue was carried out according to conditions reported by Mineno and coworkers [138].

### 4.3. C-5 Modifications

**Scheme 30:**
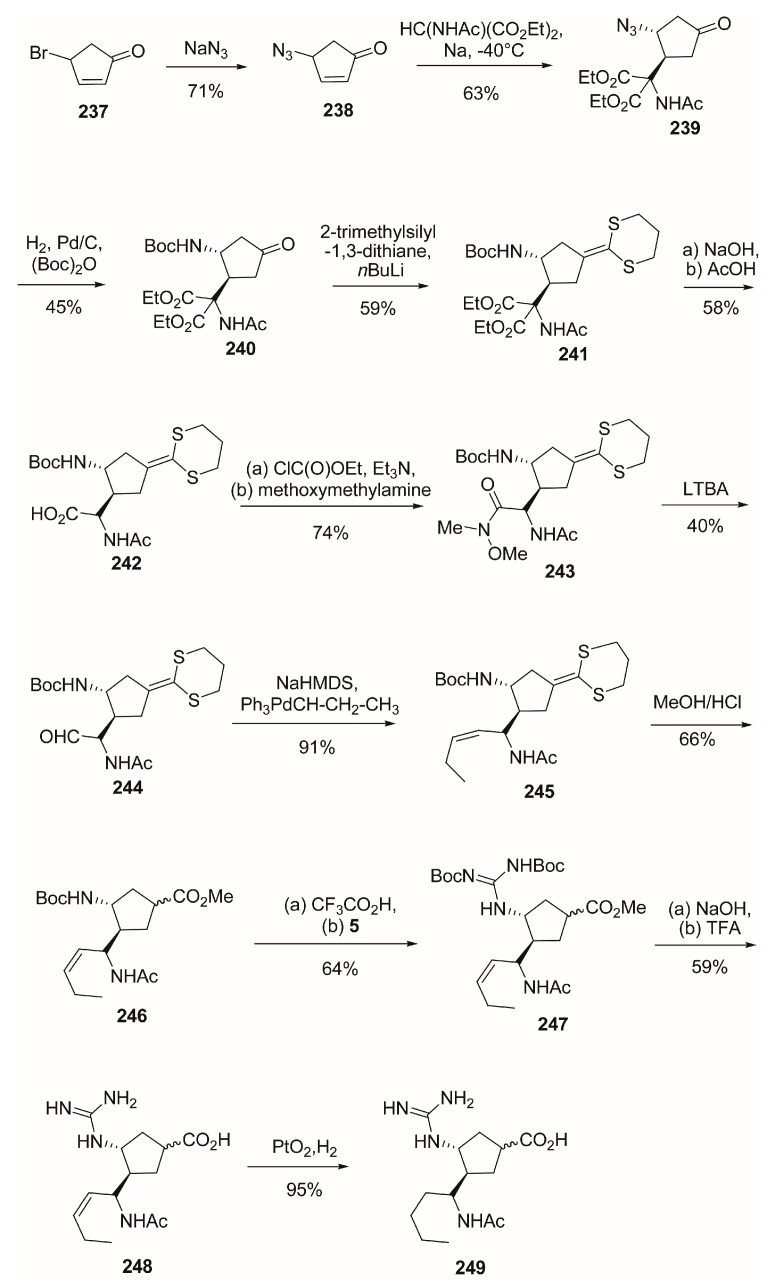
Synthetic route for peramivir analogue **249** reported by Chand and coworkers [140].

Chand and coworkers reported a synthetic approach allowing access to C-5 and C-6 modified peramivir derivatives (Scheme 30) [140]. The synthesis began with 4-bromocyclopenten-2-one (**237**), which was converted to **238** using sodium azide. Reaction of **238** with the sodium salt of diethyl acetamidomalonate in ethanol at −40 °C gave the 1,4-adduct **239**. The azido group of **239** was then converted into the Boc-protected amine **240**. Treatment of **240** with trimethysilyl 1,3-dithiane and *n*-butyllithium resulted in the formation of compound **241**. After hydrolysis of the ester groups, compound **242** was treated with ethyl chloroformate and triethylamine and then allowed to react with *N*,*O*-dimethylhydroxylamine to give methylamide **243**. Reduction of this compound with lithium tri-tert-butoxyaluminohydride (LTBA) gave aldehyde **244**, which was subjected to a Wittig reaction using propyltriphenylphosphonium bromide and sodium hexamethyl disilane (NaHMDS) to give **245**. After deprotection with methanolic HCl and TFA, the resulting amine was treated with 1,3-bis(tert-butoxycarbonyl)-2-methyl-2-thiopseudourea (**5**) in the presence of HgCl_2_ to give **247**. Hydrolysis of the methyl ester was followed by removal of the Boc groups to obtain **248**. The hydrogenation of the double bond of **248** in the presence of platinum (IV) oxide led to peramivir analogue **249**. Inhibitory activity studies revealed that the inhibitory activity of **249** was no greater than that of oseltamivir or zanamivir towards influenza A and influenza B neuraminidases (serotypes not specified).

**Scheme 31:**
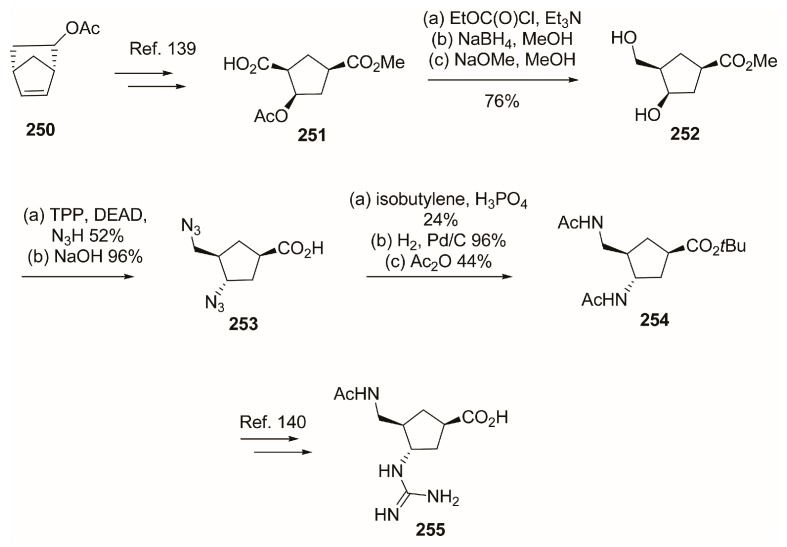
Synthetic route to peramivir analogue **255** reported by Hronowski and Szarek [141].

A synthesis of the trisubstituted cyclopentane **255** starting from 4β-acetyloxy-3β-carboxycyclopentane-1β-carboxylate (**250**) was reported by Hronowski and Szarek (Scheme 31) [141]. The carboxyl group of **250** was reduced to hydroxymethyl with sodium borohydride and the acetate group was removed with sodium methoxide in methanol. After hydrolysis of the methyl ester, both hydroxyl groups of **251** were replaced with azide groups using hydrazoic acid, diethyl azodicarboxylate (DEAD) and tetraphenylprofyrin (TPP) to give the methyl ester **253**. The reduction of the azido group was performed under hydrogen atmosphere in presence of a catalytic amount of palladium on carbon to provide the corresponding amine. The selective acetylation of the aminomethyl group was carried out by treatment with acetic anhydride at 0 °C leading to compound **254**. Guanylation and subsequent Boc deprotection was achieved as described above in Scheme 30 to obtain **255**. This compound showed low inhibitory activities in comparison with zanamivir and olsetamivir against influenza A and influenza B neuraminidases (serotypes not specified).

**Scheme 32:**
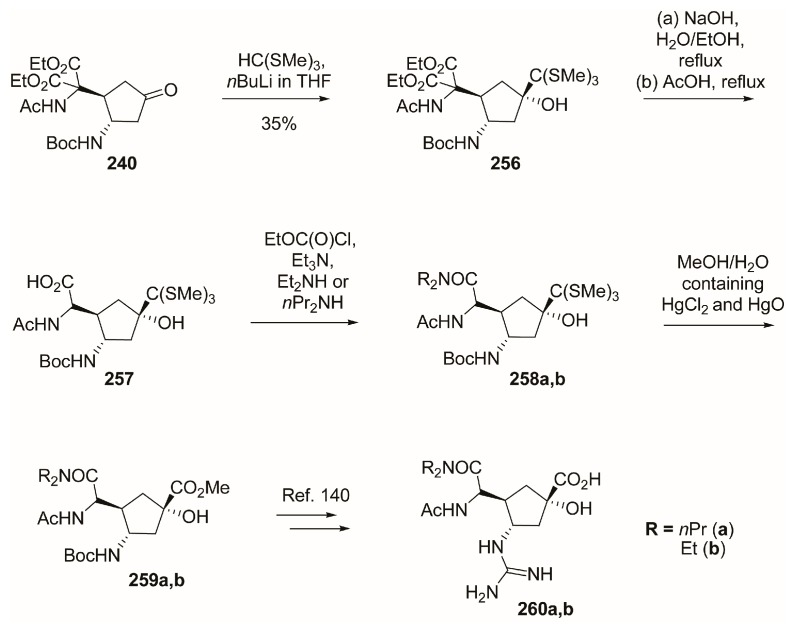
Synthetic route to peramivir analogues **261a** and **261b** reported by Chand and coworkers [142].

A stereodivergent synthesis to C-2, C-5 and C-6 modified peramivir derivatives was reported in another work by Chand and coworkers [142], using cyclopentanone **240** as starting material. For the synthesis of derivatives **260a** and **260b** (Scheme 32), attack by deprotonated tris(methylthio)methane on **240** yielded compound **256**, which was then converted to **257** by treatment with NaOH. The formation of the amide was carried out using either diethylamide or dipropylamide to obtain compounds **258a** and **258b**, respectively, which were then converted to the methyl esters **259a** and **259b** by treatment with methanol and mercury (II) chloride. After removal of the Boc groups with TFA, guanylation and ester hydrolysis were performed as described before (Scheme 30) to yield peramivir analogues **260a** and **260b**. The C-2 diastereoisomer of compound **260b** was synthesized by changing the order of the guanylation and tris(methylthio)methane addition steps. The screening of the inhibitory activities revealed that the introduction of an *N*,*N*-substituted amide on the C-5 side-chain as well as the introduction of a hydroxyl group in C-2 has an adverse effect on the inhibitory activity against influenza A neuraminidase.

**Scheme 33:**
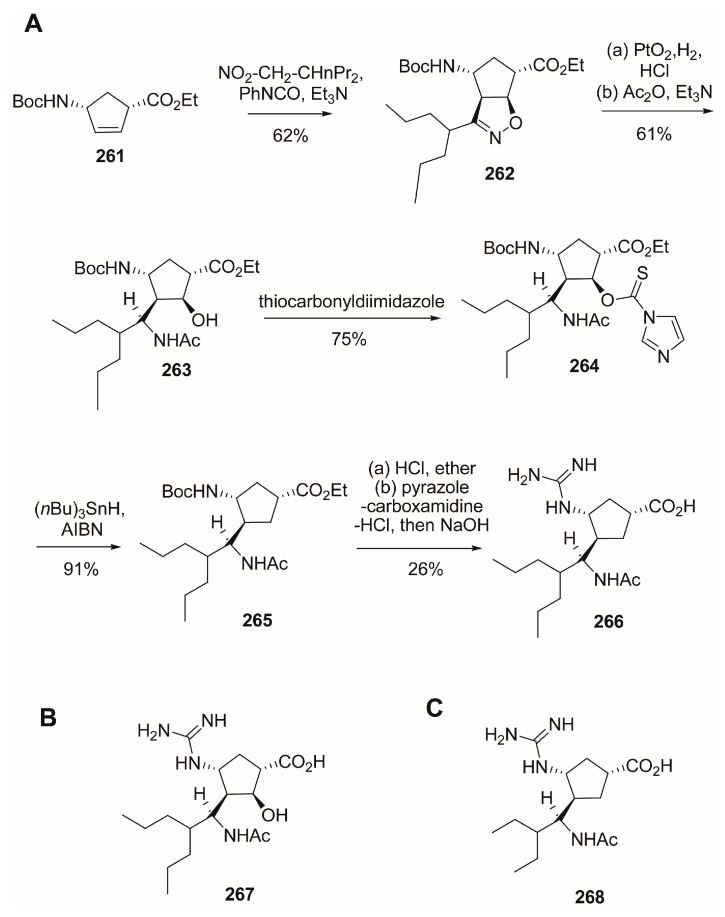
Synthesis of C-5 modified peramivir analogues. (**A**) The synthetic route to peramivir analogue **266** reported by Chand and coworkers [134]; (**B**) Peramivir derivative **267** synthetized by Chand and coworkers [134]; (**C**) Peramivir derivative **268** studied by Smee and coworkers [136].

Chand and coworkers described another synthetic route to C-5 modified peramivir analogues using an ethylated compound **227** (**261**) as starting material (Scheme 33A) [134]. The synthesis was performed in a similar way to that reported by Babu and coworkers [132]. Compound **261** was allowed to react with 1-nitro-3-*n*-propylpentane to afford **262**. This was then stirred under hydrogen atmosphere in the presence of platinum (IV) oxide to yield **263**, which was treated with thiocarbonyldiimidazole to provide **264**. 

Compound **264** was then subjected to a free radical reaction with (*n*Bu)_3_SnH and AIBN to give **265**. After deprotection, peramivir analogue **266** was obtained. Peramivir derivative **267** (Scheme 33B) was synthetized using the same synthetic strategy but maintaining the C-5 linked hydroxyl group. In vivo inhibitory activity tests of compounds **266** and **267** had similar or better inhibitory efficacy in comparison with zanamivir and oseltamivir when given orally or intranasally. In another study, Smee and coworkers studied [136] the inhibitory activity of cyclopentane derivatives **266**, **267** and **268** (Scheme 33C) [136]. All analogues showed similar inhibitory activities in comparison to peramivir and displayed greater inhibitory activity than oseltamivir or zanamivir.

## 5. Conclusions

Neuraminidase inhibitors have evolved from DANA to zanamivir through the introduction of a guanidine group to the C-4 position; from zanamivir to laninamivir by methylation of the C-7 hydroxyl group; to oseltamivir by modifying the heterocycle to a carbocycle; and from oseltamivir to peramivir by contraction of the 6-membered ring to 5-membered ring. Several of the derivatives described here showed increased inhibitory potential in comparison to their predecessor compounds. The synthesis of zanamivir has been performed either starting from a pyranose ring structure such as Neu5Ac or d-glucono-δ-lactone, or by formation of the pyranose ring through a Henry reaction or nucleophilic substitution. Among the modifications to the zanamivir core, the replacement of the carboxylic acid moiety for a phosphonate group has been demonstrated to increase inhibitory activity against H1N1, H3N2 and H5N1 influenza neuraminidases whereas the esterification of the carboxylic acid has in every case resulted in reduced inhibitory potential. To our knowledge, modifications at C-2 and C-3 have never been studied. Inhibitory activity could not be enhanced by performing modifications on C-4, C-5, C-6 or C-9, while modifications at C-7 have been shown to be capable of enhancing the inhibitory activity of zanamivir. The C-7 methoxy zanamivir derivative laninamivir showed much higher inhibitory activity against influenza B neuraminidase than zanamivir. Linking zanamivir to polymers or the formation of C-7 linked zanamivir dimers has resulted in interesting compounds with higher inhibitory activity in comparison with zanamivir. Lower inhibitory activities were detected in derivatives with modifications to the C-6-glycerol side-chain. The synthesis of oseltamivir has been thoroughly studied and five different retrosynthetic analyses have been explored. Among the derivatives of oseltamivir which have been synthesized, oseltamivir phosphonate has been demonstrated to be a significantly more potent inhibitor against H1N1 and H5N1 neuraminidases than oseltamivir. No modifications on C-2, C-3 and C-7 of the oseltamivir structure have yet been reported. The introduction of a guanidine group at the C-4 position of the hydrolyzed oseltamivir structure significantly enhanced its inhibitory activity whereas no modifications at C-5 resulted in any improvement. While C-6 modified oseltamivir analogues were synthetized, no inhibition studies of these compounds have yet been performed. Only one retrosynthetic analysis based on the use of the bicyclic compounds **226** and **232** as precursors has been developed for the synthesis of peramivir. None of the modifications performed on the peramivir scaffold could improve its inhibitory activity over peramivir itself. In contrast to zanamivir, it was reported that the lack of a guanidyl group in the peramivir structure showed little effect on its inhibitory activity. The high number of recent publications in the field of neuraminidase inhibitor synthesis reflects the huge ongoing effort to find yet more potent neuraminidase inhibitors.
